# Causal relationship between obstructive sleep apnea and diabetic nephropathy: bidirectional and multivariable Mendelian randomization study

**DOI:** 10.1080/0886022X.2025.2569086

**Published:** 2025-10-14

**Authors:** Li Zhao, Zerui Liu, Rundong Zhang, Yanan Li, Xi Zhang

**Affiliations:** ^a^ Department of Nephrology, Second Hospital of Shanxi Medical University, Taiyuan, Shanxi, China; ^b^ Department of Nephrology, Shanxi Kidney Disease Institute, Taiyuan, Shanxi, China; ^c^ Institute of Nephrology, Second Clinical Medical College, Shanxi Medical University, Taiyuan, Shanxi, China; ^d^ First Clinical Medical College, Shanxi Medical University, Jinzhong, Shanxi, China; ^e^ Department of Respiratory and Critical Care Medicine, Shanxi Medical University, Taiyuan, Shanxi, China; ^f^ Clinical Research & Innovation Unit, Xin Hua Hospital, Shanghai Jiao Tong University School of Medicine, Shanghai, China

**Keywords:** Diabetic nephropathy, CKD progression, type 2 diabetes, OSA, CPAP therapy implications, MR methodology

## Abstract

Obstructive sleep apnea (OSA) has been widely associated with DN in observational studies; however, the causal nature and direction of this association remain uncertain. To clarify this, we performed a bidirectional two-sample Mendelian randomization (MR) analysis using large-scale genetic datasets. Genetic instruments for OSA were derived from a genome-wide association study (GWAS) comprising up to 476,853 individuals, while genetic associations for DN were obtained from another GWAS including 452,280 participants. We applied multiple MR methods to ensure robust causal inference, including inverse variance weighting, MR-Egger regression, weighted median, weighted mode, and simple mode. Sensitivity analyses were thoroughly conducted using Cochran’s Q test for heterogeneity and the MR-Egger intercept test to assess potential pleiotropy. Furthermore, multivariable MR was employed to evaluate the independent effect of OSA on DN after adjusting for hyperlipidemia and hypertension. The results indicated a significant causal effect of OSA on DN risk, supported by IVW (OR = 1.41, 95% CI: 1.12–1.77, *p* = 0.003) and weighted median estimates (OR = 1.57, 95% CI: 1.16–2.13, *p* = 0.003). Reverse MR analysis revealed no evidence of a causal effect of DN on OSA. Importantly, after accounting for hyperlipidemia and hypertension, multivariable MR confirmed that OSA exerts an independent causal influence on DN (OR = 0.90, 95% CI: 0.14–1.67, *p* = 0.021). These findings suggest that OSA may contribute to the pathogenesis of diabetic nephropathy through specific mechanisms, independent of traditional metabolic risk factors.

## Graphical abstract

**Figure:**
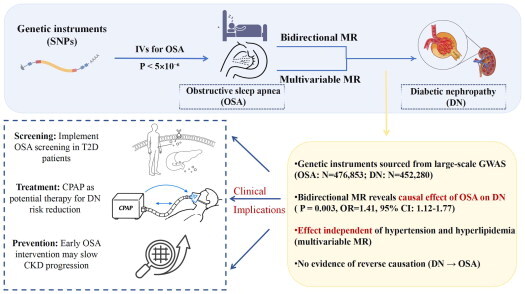

## Introduction

Diabetic nephropathy (DN) is a leading cause of end-stage renal disease (ESKD) [1] and poses significant challenges to global health systems. Elevated albuminuria and reduced estimated glomerular filtration rate (eGFR) are independently associated with increased risks of all-cause mortality and cardiovascular-related mortality [2,3]. While comprehensive management, including glycemic, blood pressure, and lipid control, can slow the progression of DN [4], many patients inevitably progress to ESKD [5]. This underscores the urgent need to identify additional pathogenic factors to develop more effective prevention and treatment strategies for DN. In this study, DN cases were defined according to standardized clinical criteria, including persistent albuminuria (UACR > =30 mg/g) and/or reduced estimated glomerular filtration rate (eGFR <60 mL/min/1.73m^2^) in individuals with type 2 diabetes, consistent with established clinical criteria widely adopted in contemporary research [6,7].

Obstructive sleep apnea (OSA) is increasingly recognized as a potential contributor to the pathogenesis of diabetes-related complications. Epidemiological studies have reported a strong association between OSA and type 2 diabetes mellitus (T2DM), with a notably high prevalence of OSA among individuals with T2DM [8]. Moreover, patients with concurrent OSA and T2DM are more likely to develop the diabetic peripheral neuropathy (DPN) [9]. Additionally, OSA has been linked to heightened oxidative and nitrosative stress and impaired microvascular regulation in T2DM [9], and then exacerbate the onset and progression of microvascular complications and finally leading DN. While one Mendelian Randomization study has established a causal link between OSA and diabetic microangiopathy, the potential causal relationship between OSA and DN remains unclear [10].

Therefore, we conducted a Mendelian randomization (MR) analysis using single nucleotide polymorphisms (SNPs) as instrumental variables (IVs) to explore the causal association between OSA and DN. By leveraging genetic variants as “natural” randomized experiments, MR minimizes biases from confounding variables and reverse causation [11,12]. This study utilized GWAS data to provide insights into potential strategies for prevention and managing DN.

## Methods

### Univariable Mendelian randomization

#### Study design

**Figure 1.**
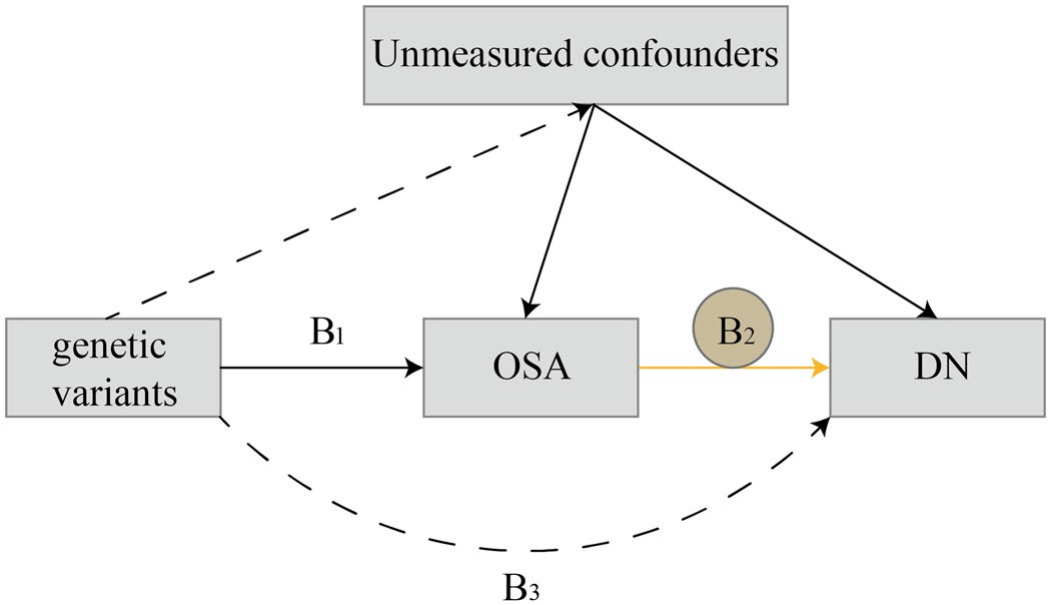
Key assumptions in the Mendelian randomization models. Hypothesis 1: IVs were associated with the exposure factor; hypothesis 2: IVs were independent of confounding factors; hypothesis 3: IVs influenced the outcome only through the exposure factor. B_2_ indicates the causal relationship of interest to be estimated, where B_2_=B_3_/B_1_. B_1_, and B_3_ represent estimated direct effects of a genetic variant on the OSA and CKD, respectively. Solid paths are theorized to exist; dashed paths are theorized to be nonsignificant according to MR assumptions.

We conducted a two-sample MR analysis to explore the potential causal relationship between OSA and DN. Additionally, we conducted a reverse MR analysis to evaluate the causality in the converse direction, from DN to OSA. The MR approach leverages genetic variants as instrumental variables (IVs) to infer causality, minimizing bias from confounding factors [13]. The validity of MR relies on three core assumptions: (1) the genetic instruments are strongly associated with the exposure; (2) the genetic instruments are not associated with any confounders of the exposure-outcome relationship; and (3) the genetic instruments influence the outcome only through the exposure (Figure 1) [14]. We report this MR study with reference to STROBE-MR (Strengthening the Reporting of Observational Studies in Epidemiology Using Mendelian Randomization) [15].

#### Data source

Summary-level genome-wide association study (GWAS) data for DN were obtained from the GWAS Catalog (https://www.ebi.ac.uk/gwas/), involving 452,280 individuals of European ancestry (1,032 cases and 451,248 controls). For OSA, GWAS summary statistics (ebi-a-GCST90018916) was based on 476,853 individuals, including 13,818 cases and 463,035 controls, identifying 24,183,940 single nucleotide polymorphisms (SNPs). All datasets were sourced from publicly available GWAS repositories (https://gwas.mrcieu.ac.uk/), and all studies received approval from their respective institutional review boards. Diabetic nephropathy cases were defined per the GWAS Catalog study GCST90018832, requiring either: persistent albuminuria (UACR> =30 mg/g), or eGFR <60 mL/min/1.73m^2^ in diabetic patients [6,16].

To minimize the bias from population stratification, both exposure and outcome datasets were restricted to individuals of European descent.

### Selection of IVs for DN and OSA

SNPs exhibiting genome-wide significant significance (*p* < 5 × 10^−6^) were selected as IVs, consistent with prior statistical guidelines [14]. To ensure the independence of IVs, linkage disequilibrium (LD) thresholds were set at r^2^ < 0.001 and a distance of 10,000 kb [17]. SNPs in LD were excluded from further analyses. In the instrument selection process, the cumulative R^2^ values were calculated as 0.072 for OSA-associated SNPs and 0.627 for DN-associated SNPs, representing the proportion of phenotypic variance explained by the respective genetic instruments.

To mitigate weak instrumental bias, SNPs with an F-statistic >20 was retained, ensuring strong instrument strength [18]. All selected OSA-associated genetic instruments demonstrated strong association strength (F-statistic range: 479.67–2839.38, median = 922.87), with all meeting the recommended threshold criterion of *F* > 20. All selected DN-associated genetic instruments showed robust association strength (F-statistic range: 5241.15–72974.42, median = 12754.8986), with all exceeding the recommended threshold of *F* > 20. Palindromic SNPs were excluded to enhance precision and reliability. These criteria were uniformly applied to both forward and reverse MR analyses (refer to Table S1 and Table S2 for details).

### Statistical analysis

A suite of MR approaches was employed to examine the causal relationship between OSA and DN, including inverse variance weighting (IVW), MR-Egger, weighted median, weighted mode, and simple mode analyses. Among these, IVW, regarded as the most robust, served as the primary analytical method, while the others provided complementary insights.

Visual tools such as funnel plots and scatter plots were used to qualitatively assess symmetry and evaluate the effects. Heterogeneity among IVs was assessed using Cochran’s Q test, with *p* < 0.05 indicating significant heterogeneity. Horizontal pleiotropy was evaluated using the MR-Egger intercept test, with a P-intercept value < 0.05 suggesting its presence.

To further ensure robustness of findings, a leave-one-out analysis was performed to determine if any single SNP had a disproportionate influence on the results. All statistical analyses were conducted using R software (version 4.4.3, http://www.Rproject.org) and the “TwoSampleMR” package (version 0.5.11).

#### Multivariable Mendelian randomization

To investigate whether OSA exhibits an independent causal effect on DN after accounting for potential pleiotropic pathways, we performed multivariable Mendelian randomization (MVMR) analysis [19]. This approach enables simultaneous assessment of multiple risk factors while controlling for correlated genetic instruments [20]. Specifically, we integrated genetic variants associated with OSA, hypertension, and hyperlipidemia to evaluate their distinct effects on DN risk, ensuring compliance with core instrumental variable assumptions. The analysis was designed to minimize bias from horizontal pleiotropy by explicitly modeling shared genetic influences among exposures.

The genetic association data for OSA and DN in the multivariable MR analysis were obtained from the same sources as those used in the univariable MR analysis (detailed in the “Data Source” and “Selection of IVs” sections). For hyperlipidemia, GWAS summary statistics (ebi-a-GCST90104003) was based on 349,222 individuals, including 3,838 cases and 345,384 controls, identifying 14,502,301 SNPs. For hypertension, GWAS summary statistics (ebi-a-GCST90038608) was based on 484,598 individuals, including 2,287 cases and 482,311 controls, identifying 9,587,836 SNPs. All dataset were sourced from publicly available GWAS repositories (https://gwas.mrcieu.ac.uk/), and all studies received approval from their respective institutional review boards.

The instrumental variables (IVs) were selected following identical criteria, including genome-wide significance thresholds (*p* < 5 × 10^−6^), linkage disequilibrium parameters (r^2^ < 0.001, kb = 10,000), and F-statistic filtering (*F* > 10) to ensure robustness.

#### Statistical power calculation

To ensure our Mendelian randomization analysis was sufficiently powered to detect a clinically relevant effect size, we performed *a priori* statistical power calculations using R statistical software (version 4.4.3) [21], based on established methods for binary outcomes in Mendelian randomization studies [22].

The statistical power was derived using the formula: Power = Φ(√(N × R^2^ × (β)^^2^/var(Y)) - z_{α/2}), where Φ denotes the cumulative distribution function of the standard normal distribution; N is the total sample size of the outcome dataset; R^2^ represents the proportion of variance in the exposure variable explained by the genetic instrument(s); β is the assumed log odds ratio per standard deviation increase in the exposure; var(Y) approximates the variance of the binary outcome; and z_{α/2} is the critical value for a two-sided test at significance level α (set to 0.05). The parameters utilized in our calculation were directly derived from the study data: Causal effect (β): ln(1.41) ≈ 0.344; Outcome sample size (N): 452,280; Number of cases: 1,032; Variance explained (R^2^) by OSA instruments: 0.0724; Significance level (α): 0.05.

## Results

### Causal effect of OSA on DN

The two-sample MR analyses have demonstrated a potential causal relationship between genetically predicted OSA and the susceptibility to DN, utilizing a panel of 32 SNPs as genetic instruments. The IVW method yielded an odds ratio (OR) of 1.41 (95% confidence interval [CI]: 1.12–1.77, *p* = 0.003), indicating a significant positive association between OSA and DN. Concordantly, the weighted median method also supported this causal link with an OR of 1.57 (95% CI: 1.16–2.13, *p* = 0.003). The OR values (1.41–1.57) demonstrate that patients with OSA exhibit a 41–57% increased risk of diabetic nephropathy incidence relative to those without OSA.

**Figure 2.**
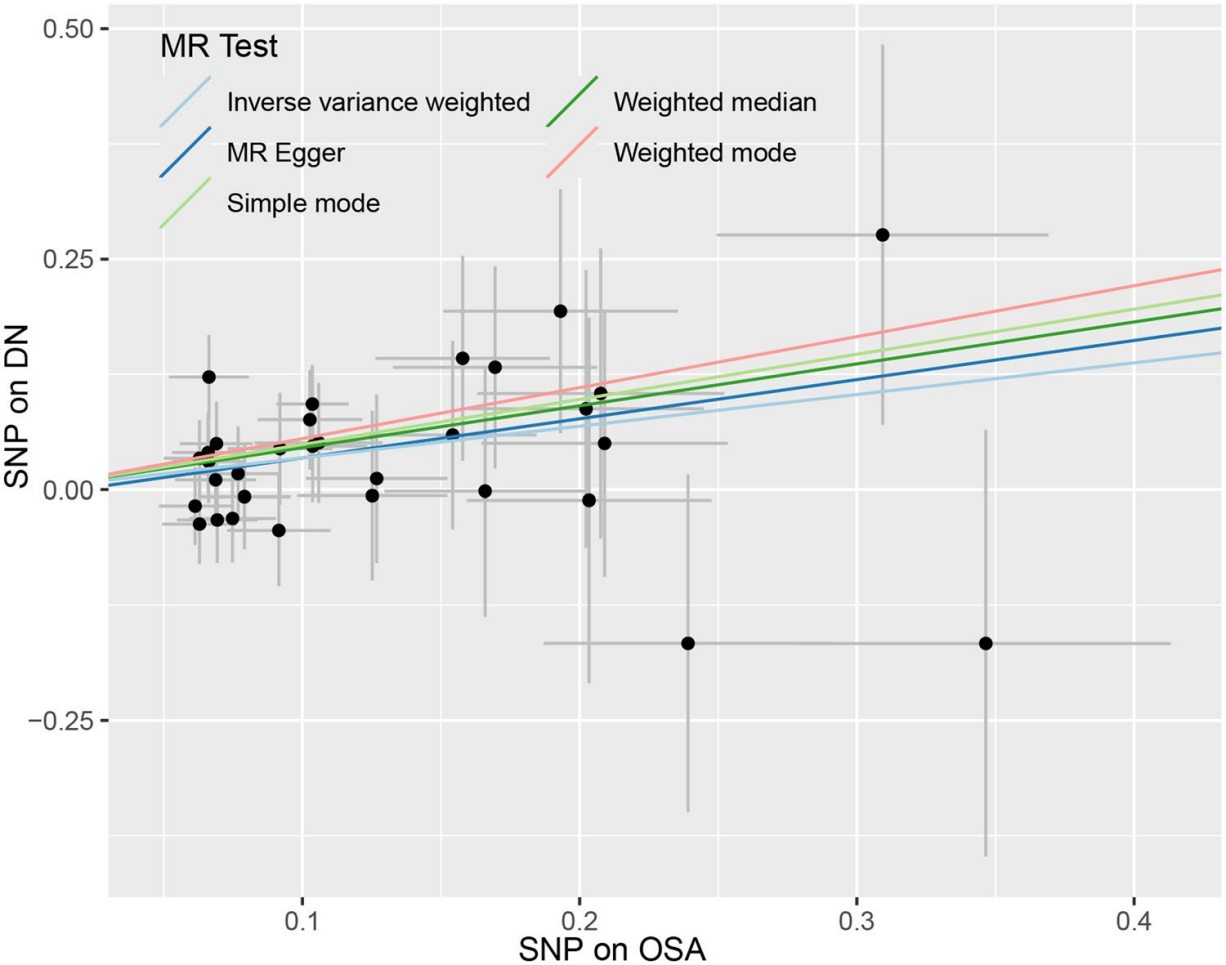
Scatterplot of SNP potential effects on OSA vs. DN. The slope of each line corresponding to estimated MR effect per method.

**Figure 3.**
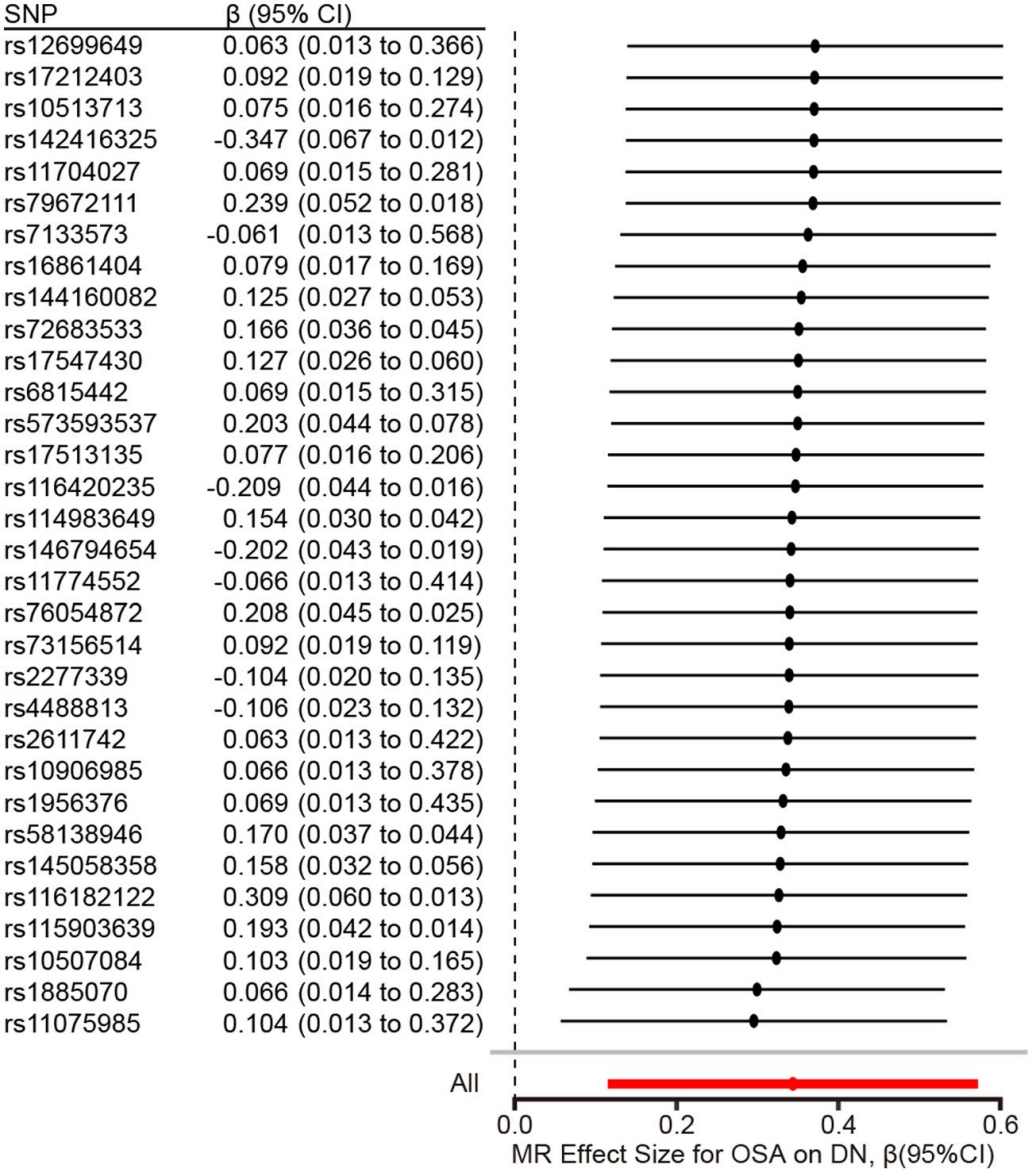
MR-estimated effect sizes of each OSA-related SNP on DN.

**Figure 4.**
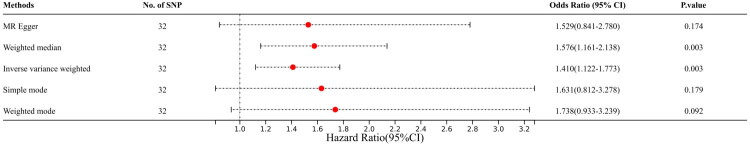
Causal relationship between OSA and DN are shown by forward Forest plot.

Notwithstanding, the remaining three MR methods did not affirm a causal connection between OSA and DN. The scatter plot and forest plot visualizations provided in Figures 2 and 3 further illustrate these findings. The Cochran’s Q test within the IVW framework (*Q* = 22.352, *p* = 0.872) and the MR-Egger regression (*Q* = 22.270, *p* = 0.844) both indicated a lack of heterogeneity among the instrumental variables, thereby enhancing the robustness of the primary findings. Additionally, the MR-Egger intercept analysis negated the presence of directional pleiotropy (*p* = 0.776), suggesting that the observed effects are likely not due to confounding pleiotropic influences. Additionally, the MR-PRESSO analysis also showed no evidence of heterogeneity (global test *p* = 0.882 > 0.05), further confirming the robustness of our instrumental variable assumptions. Lastly, sensitivity analyses involving leave-one-out procedures confirmed the stability of the observed association, as the removal of any individual SNP did not significantly alter the overall results, as depicted in Figure 4. Visual representation of the funnel plot is available in Supplementary Figures S4

**Figure 5.**
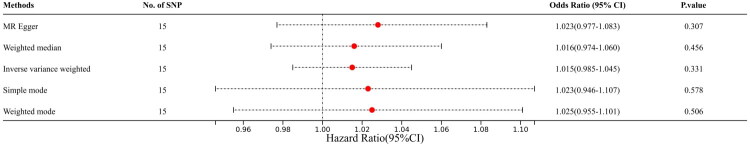
Causal relationship between DN and OSA are shown by reverse Forest plots.

To assess the robustness of the primary findings, we employed a suite of complementary MR methods, each with distinct underlying assumptions. The consistency in the direction of effect across all methods (OR > 1) strengthens the credibility of the causal inference. Specifically, the weighted mode method provides a consistent causal estimate when the plurality of genetic variants are valid instruments, and it is particularly influenced by variants with larger weights (i.e. stronger associations with the exposure) [15]. In contrast, the simple mode estimate is more susceptible to the influence of a larger number of genetic variants with smaller individual effects. The fact that both mode-based estimates, despite their differing sensitivities, aligned in direction with the more precise IVW estimate supports the conclusion that our results are not driven by a few outliers or invalid instruments.

We performed a comprehensive statistical power analysis based on the following parameters: observed odds ratio (OR = 1.41), exposure (OSA) GWAS sample size (*N* = 452,280), cumulative R^2^ = 0.072387, outcome case number (n cases = 1,032), and total sample size (*N* = 452,280). The analysis demonstrated that our study achieved a statistical power of 95.3% (non-centrality parameter, NCP = 13.21), with an exceptionally strong instrument strength (F-statistic = 37,211.59 > 20). These results confirm that our study was well-powered (>80% threshold) to detect the target effect size of clinical relevance.

Given the highly polygenic nature of OSA and the limited number of genetic variants meeting the genome-wide significance threshold (*p* < 5 × 10^−8^) (only one SNP was identified), we adopted a relaxed threshold (*p* < 5 × 10^−6^) to ensure sufficient instrumental variables for Mendelian randomization analysis. To validate the robustness of our findings, we conducted a supplementary Wald ratio analysis using the sole SNP (rs11075985) that met the standard threshold (*p* < 5 × 10^−8^). Results indicated a positive association between OSA and DN (beta = 0.896, SE = 0.411, *p* = 0.029), with consistent directionality compared to the primary analysis, further supporting the potential causal role of OSA in increasing DN risk. Although the precision decreased due to the reduced number of instruments, the concordance in effect direction strengthens the reliability of our findings. Detailed results are provided in Supplementary Table S3 and Figure S5.

### Causal effect of DN on OSA

IVW analysis revealed no causal relationship between DN and OSA, with an OR of 1.01 with 95% CI of 0.89 to 1.12 (*p* = 0.331) (refer to Figure S1 for details), which was reconfirmed by the findings from other four MR methods. Both MR-Egger regression analyses yielded non-significant p-values (*p* = 0.543), indicating the absence of potential pleiotropic effects influencing the results. Additionally, the Cochran’s Q tests conducted within both the IVW (*Q* = 11.440, *p* = 0.651) and MR-Egger (*Q* = 11.050, *p* = 0.607) frameworks suggested a lack of heterogeneity among the IVs, thereby reinforcing the reliability of the obtained findings. Furthermore, sensitivity analyses employing leave-one-out procedures demonstrated that no individual SNP significantly impacted the overall estimated effect of OSA on diabetic nephropathy. The minimum F-statistic value is provided in Supplementary Table S2 for reference. Visual representations of the leave-one-out plot and funnel plot are available in Supplementary Figures S2 and S3, respectively, offering additional insights into the stability and precision of the MR analyses conducted in this study. Figure 5 demonstrates no statistically significant causal relationship between DN and OSA across multiple Mendelian randomization methods.

### Multivariable MR

The MVMR analysis demonstrated a statistically significant independent causal association between genetically predicted OSA and DN after adjusting for hyperlipidemia and hypertension (Table 1). Using the multivariable IVW method, OSA showed a significant positive causal effect on DN risk (OR = 2.47, 95% CI: 1.15–5.32, *p* = 0.021 < 0.05), while neither hyperlipidemia (OR = 0.93, 95% CI: 0.84–1.03, *p* = 0.154) nor hypertension (OR = 8.38 × 10–15, 95% CI: 6.20 × 10–54 − 1.13 × 10^25^, *p* = 0.481) exhibited significant associations. These findings were corroborated by sensitivity analyses using multivariable MR-Egger (OSA on DN, OR = 2.85, 95% CI: 1.04–7.80, *p* = 0.042 < 0.05) and multivariable median methods (OSA on DN, OR = 2.55, 95% CI: 1.060–6.153, *p* = 0.037 < 0.05), supporting the robustness of the observed OSA-DN relationship.

**Table 1.** Multivariable Mendelian randomization results.

Exposure	Outcome	IVW		Multivariable MR-Egger		Multivariable median	
		OR (95% CI)	*p*-value	OR (95% CI)	*p*-value	OR (95% CI)	*p*-value
OSA	DN	2.47 (0.15-5.32)	0.021	2.85 (1.04-7.80)	0.042	2.55 (1.06-6.15)	0.037
hypertension	DN	8.38e-15 (6.20e^-54^-1.13e^25^)	0.481	1.22e-16 (e^-131^-e^58^)	0.452	1.38e-12 (e^-149^-e^95^)	0.665
hyperlipidemia	DN	0.93 (0.84-1.03)	0.154	0.94 (0.83-1.05)	0.251	0.92 (0.79-1.07)	0.279

However, we must emphasize that due to the inherent limitations of GWAS datasets, unmeasured clinical factors such as diabetes duration, glycemic control levels, and other diabetes-related complications may still introduce residual confounding effects, potentially influencing the observed associations in this study.

## Discussion

Although the IVW method demonstrated a significant association between OSA and DN, it should be noted that only two out of five MR methods reached statistical significance. This inconsistency likely reflects methodological differences among the approaches. Specifically, IVW, as a method that does not adjust for pleiotropy, typically maintains the highest statistical power, whereas MR-Egger suffers from approximately 50% power reduction due to the requirement of estimating an intercept term [23]. Furthermore, the non-significant results from simple mode and weighted mode methods likely reflect their inherently lower statistical power compared to IVW, as these approaches are more conservative and less efficient for detecting modest causal effects. Despite these methodological variations, the consistent effect direction across all methods (OR > 1) provides some support for the robustness of the observed association.

Based on the current findings, we cautiously suggest that OSA may potentially exhibit a causal relationship with DN, though this interpretation requires careful consideration. The methodological discrepancies may indicate residual pleiotropic bias that has not been fully accounted for. Additionally, the predominantly European ancestry of the GWAS samples may limit the generalizability of our results. Future research should prioritize validation through more ancestrally diverse cohorts, application of advanced MR methodologies like MR-PRESSO, and integration with prospective cohort study designs to strengthen causal inference. These approaches would help address current limitations while providing more robust evidence regarding the OSA-DN relationship.

The latest research has shown a strong connection between genetically determined OSA and a higher chance of developing DN. To our knowledge, this represents the inaugural study to examine the causality between DN and OSA by employing a bidirectional two-sample MR approach. In this inquiry, by leveraging openly available aggregated statistical data, we did not discover convincing proof that a genetic predisposition to DN elevates the risk of OSA. Conversely, MR findings indicated that OSA is linked to an augmented risk of DN, unveiling a fresh avenue for prospective clinical treatments aimed at individuals suffering from diabetic nephropathy. The OR values (1.41–1.57) demonstrate that patients with OSA exhibit a 41–57% increased risk of diabetic nephropathy incidence relative to those without OSA. The observed 41–57% increased risk of diabetic nephropathy among obstructive sleep apnea patients carries important public health significance. Given the high prevalence of OSA in diabetic populations and the progressive nature of diabetic kidney disease, even this moderate elevation in relative risk could translate to substantial numbers of preventable cases at the population level. The potential for risk modification through existing OSA therapies (such as CPAP treatment) further underscores the public health relevance of these findings. These results suggest that improved OSA screening and management in diabetic populations could have meaningful impacts on reducing the burden of diabetic nephropathy. From a clinical perspective, these findings highlight several important considerations. First, they reinforce the value of routine OSA screening in diabetic patients, particularly those with additional risk factors for kidney disease. Second, they suggest that optimal management of OSA may represent a novel approach to preventing or slowing the progression of diabetic nephropathy, although this requires confirmation through intervention studies. Finally, these results emphasize the need for heightened vigilance for early signs of kidney disease in diabetic patients with comorbid OSA.

The multivariable MR analysis further strengthens the causal inference between OSA and DN by accounting for potential confounding from cardiometabolic comorbidities. After adjusting for hyperlipidemia and hypertension, OSA retained a statistically significant independent effect on DN risk, while neither comorbidity showed significant associations. This suggests that the OSA-DN relationship is unlikely to be fully explained by shared pathways with these metabolic conditions. The consistency across IVW, MR-Egger, and weighted median methods reinforces the robustness of this finding. However, residual confounding from unmeasured factors cannot be entirely ruled out, highlighting the need for future studies with richer clinical data to validate these observations.

To our knowledge, this represents the first Mendelian randomization study to comprehensively investigate the bidirectional causal relationship between OSA and DN while simultaneously accounting for key cardiometabolic confounders *via* multivariable MR analysis. This approach distinguishes our work from the recent Mendelian randomization study by Liu et al. that examined the association between OSA and a broad phenotype of diabetic microangiopathy [10], allowing us to specifically isolate the independent effect of OSA on diabetic kidney disease. Past studies have generally agreed that OSA is closely associated with the onset of DN. For example, a systematic review and meta-analysis has elucidated that chronic intermittent hypoxemia, which is characteristic of OSA, leads to the activation of oxidative stress and inflammatory pathways. These pathways have been implicated in the pathogenesis of diabetes-related microvascular complications, including DN [24]. A cohort study has indicated that patients with OSA and type 2 diabetes are more likely to have DN compared with those with type 2 diabetes but without OSA [9]. Additionally, many studies have demonstrated a strong association between OSA and the development of complications in DN. For instance, a large-scale cohort study revealed that, compared to T2D patients without OSA, those with comorbid OSA exhibited an elevated risk of cardiovascular disease, atrial fibrillation, peripheral neuropathy, diabetes-related foot complications, chronic kidney disease, and all-cause mortality. A cross-sectional study found a significantly higher prevalence of neuropathy among OSA patients compared to non-OSA individuals, with OSA remaining independently associated with diabetic neuropathy [9]. Another cohort study proposed severe OSA as a risk factor for diabetic macular edema [25].

Our study provides robust genetic evidence positioning OSA not merely as an association, but as a causal and targetable upstream driver in the network of diabetic complications, specifically DN. This finding fundamentally challenges the prevailing clinical paradigm, which has long been constrained to a narrow set of modifiable risk factors-glycemic, blood pressure, and lipid control-leaving an unmet need for effective prevention strategies as many patients continue to progress to end-stage renal disease despite optimal management of these parameters [26,27]. The identification of OSA as an independent risk factor introduces a novel, actionable pathway for intervention. Crucially, unlike genetic predispositions, OSA is a highly prevalent and modifiable condition with established diagnostic and therapeutic protocols. This implies that systematic screening for OSA in diabetic populations - particularly those with additional risk factors or early signs of kidney disease - could identify a high-risk subgroup amenable to targeted therapy. Indeed, existing studies suggest that interventions such as continuous positive airway pressure (CPAP) or weight loss [28], which ameliorate OSA, may concurrently reduce oxidative stress, improve endothelial function, and mitigate nocturnal hypoxia - key mechanistic pathways linking OSA to microvascular damage. For instance, CPAP therapy has been shown to reduce biomarkers of nitrosative stress and improve glycemic variability, which could theoretically translate into renal protection [6,9]. Collectively, these interventional studies provide mechanistic support for our genetic findings, suggesting that targeted treatment of OSA may translate into tangible risk reduction for diabetic nephropathy. Therefore, treating OSA extends beyond improving sleep quality; it represents a paradigm shift toward multi-system risk modulation in diabetes care, offering a complementary strategy to conventional therapies to potentially slow or prevent the onset of DN. Future prospective studies and randomized trials are warranted to confirm whether OSA treatment directly reduces DN incidence, but our findings underscore the urgency of integrating sleep medicine into routine diabetes management. The clinical implications of these causal findings are summarized in Table 2, highlighting potential translational applications for screening, treatment, and prevention strategies in diabetic kidney disease.

**Table 2.** Clinical implications of the causal relationship between OSA and DN.

Domain	Current challenge	Implication from Our study	Potential action
Screening	DN screening is focused on urinary albumin excretion and eGFR decline. There is no routine screening for novel, modifiable risk factors beyond glucose/BP/lipids [26,27].	OSA is an independent, causal, and modifiable risk factor for DN.	Implement routine OSA screening in patients with type 2 diabetes, especially those with poor glycemic control, obesity, or hypertension. Use polysomnography for high-risk individuals.
Treatment	Standard DN treatment relies on renin-angiotensin-aldosterone system (RAAS) blockade, SGLT2 inhibitors, and strict metabolic control [29].	Treating OSA may represent a novel, complementary therapeutic pathway to slow DN progression by mitigating hypoxia, oxidative stress, and inflammation.	Prioritize OSA treatment (CPAP, mandibular advancement devices) in diabetic patients diagnosed with OSA [9]. Clinical trials are needed to test if OSA treatment directly reduces albuminuria or slows eGFR decline.
Prevention	Primary prevention of DN is limited to optimizing glycemic and blood pressure control from the onset of diabetes [26,27].	Identifying and treating OSA early in the course of diabetes could potentially prevent or delay the onset of kidney damage.	Integrate sleep health into multidisciplinary diabetes care teams.

The causal relationship identified in our Mendelian randomization analysis is further bolstered by its concordance with the mechanistic understanding of OSA pathophysiology and finds relevance in the context of major contemporary interventional trials. Large-scale randomized controlled trials, such as the SAVE (Sleep Apnea Cardiovascular Endpoints) trial [30], were primarily designed to investigate the impact of CPAP therapy on cardiovascular events in patients with established cardiovascular disease and moderate-to-severe OSA. While the SAVE trial did not demonstrate a significant reduction in the primary composite cardiovascular endpoint, subsequent exploratory analyses have suggested potential benefits in certain subgroups and for other outcomes [31]. Notably, renal endpoints were not a pre-specified focus of these major trials. Our genetic evidence, which establishes a direct causal link between OSA and diabetic nephropathy independent of traditional risk factors, provides a powerful rationale for post-hoc analyses of existing trial datasets to investigate the effect of CPAP on renal parameters.

In the current dialogue exploring the association between OSA and DN, it is essential to delve into the underlying pathophysiological mechanisms that may bridge these two disorders. OSA is marked by repetitive episodes of upper airway collapse during sleep, leading to intermittent hypoxemia and reoxygenation cycles. These occurrences initiate a series of oxidative stress and inflammatory cascades, which have been linked to the etiology of microvascular complications in diabetes. Specifically, the activation of these pathways can result in endothelial dysfunction, augmented vascular permeability, and the accrual of advanced glycation end-products, all contributing factors to the pathogenesis of DN [32]. Moreover, prior research has indicated that OSA, with its characteristic pattern of recurrent hypoxemia followed by reoxygenation, emulates ischemia-reperfusion injury [33]. This process elevates levels of reactive oxygen species in individuals with type 2 diabetes (T2D), intensifying oxidative and nitrosative stress. A study has demonstrated that OSA, the apnea-hypopnea index, and nocturnal hypoxemia are independently associated with increased serum nitrotyrosine levels in T2D patients, even after adjusting for confounding variables [34]. Notably, nitrotyrosine has been implicated in the pathogenesis of DN in both rodent models [35] and cell culture [29] studies, with enhanced nitrotyrosine staining observed in the renal proximal tubules and thin limbs of the loop of Henle in DN patients. Furthermore, attenuation of nitrosative stress has been shown to ameliorate DN outcomes in diabetic rodent models [36,37]. Consequently, nitrosative stress may emerge as a pivotal pathogenic link connecting OSA, T2D, and DN, underscoring the importance of targeting this pathway in the development of novel therapeutic strategies for these interrelated conditions. While the current study is limited by the lack of available GWAS data on oxidative/nitrosative stress biomarkers, we plan to conduct future research incorporating these molecular mediators once suitable genetic datasets become available. This will allow us to perform formal mediation analyses to empirically validate the proposed biological pathways linking OSA and DN.

Our Mendelian randomization findings are strongly supported by a well-characterized pathophysiological pathway that mechanistically links OSA to diabetic nephropathy. This cascade is initiated by the recurrent upper airway collapse that defines OSA, leading directly to chronic intermittent hypoxia (CIH) and sleep fragmentation [38,39]. CIH, in turn, acts as the primary driver of oxidative and nitrosative stress through multiple mechanisms: it induces mitochondrial dysfunction, activates nicotinamide adenine dinucleotide phosphate oxidases, and facilitates the uncoupling of nitric oxide synthase, which shifts its activity from producing nitric oxide (NO) to generating reactive oxygen species and peroxynitrite (ONOO-) [38,40]. The resultant state of heightened oxidative and nitrosative stress is a critical mediator of microvascular injury; it promotes endothelial dysfunction, increases vascular permeability, triggers a pro-inflammatory state, and leads to the accumulation of advanced glycation end-products [41]. Within the specific context of the diabetic kidney, this generalized microvascular damage manifests as podocyte injury, mesangial expansion, extracellular matrix accumulation, and ultimately, the classic pathological features of DN - albuminuria and a declining glomerular filtration rate. Thus, the intermittent hypoxia characteristic of OSA provides a plausible and compelling biological mechanism that connects disordered sleep to the progression of diabetic kidney disease, directly through the conduit of oxidative and nitrosative stress [42].

Our genetic evidence supporting OSA as a causal risk factor for DN aligns with and extends a growing body of literature implicating OSA in the pathogenesis of other diabetic microvascular and macrovascular complications. For instance, robust epidemiological and mechanistic studies have established strong links between OSA and diabetic retinopathy, with severe OSA identified as an independent risk factor for diabetic macular edema [43,44]. Similarly, OSA has been consistently associated with diabetic peripheral neuropathy, potentially mediated through shared pathways of oxidative stress, nitrosative stress, and impaired microvascular regulation [45]. Beyond microvascular disease, compelling evidence from large-scale studies links OSA to an elevated risk of cardiovascular diseases in patients with type 2 diabetes, including myocardial infarction, stroke, and atrial fibrillation [46]. This convergent evidence across complication types suggests that OSA may act as a systemic amplifier of diabetic damage, exacerbating injury through hypoxia-induced pathways that affect multiple organ systems simultaneously. The demonstration of a causal role for OSA in DN thus positions it as a novel, modifiable therapeutic target not only for nephropathy but potentially for the broader spectrum of diabetes-related complications. This perspective argues for integrating OSA management into a holistic approach to diabetes care, potentially offering benefits beyond improving sleep quality to encompassing protection against multiple end-organ damages.

Additionally, it is conceivable that diabetic microvascular complications may contribute to or intensify OSA. Diabetes-related microvascular complications frequently co-occur, suggesting a potential interplay among these conditions. Individuals with DN may also suffer from diabetic neuropathy, which can compromise the autonomic nervous system’s control over the pharyngeal muscles, thereby increasing susceptibility to airway collapse. Case studies have demonstrated a strong correlation between OSA and hereditary motor and sensory neuropathy diseases, such as Charcot-Marie-Tooth disease. Similarly, patients with diabetic autonomic neuropathy have been shown to have an elevated risk of developing OSA compared to those without this condition [45] https://pubmed.ncbi.nlm.nih.gov/17347559/. These findings imply a bidirectional relationship between OSA and DN. Interestingly, our study did not yield any evidence of a relationship between the two diseases.

Our MR analysis boasts several strengths. Firstly, we utilized a bidirectional MR, which helps to mitigate confounding factors and reverse causation. Secondly, this research employed data sourced from a substantial genomic research cohort comprising around 476,853 participants, ensuring considerable statistical power. Thirdly, the study’s conclusions were drawn from genetic instrumental variables, with causal relationships inferred using a variety of robust MR techniques that are impervious to horizontal pleiotropy and other potential confounders.

Our study identified several limitations that need to be addressed. Despite numerous sensitivity analyses, a thorough evaluation of horizontal pleiotropy remains challenging. The lack of individual-level data also prevented us from conducting stratified population analyses. Additionally, focusing solely on European databases limits the generalizability of our findings to other ethnic groups. Notably, the limited number of diabetic nephropathy cases (*n* = 1,032) in our GWAS source population (0.23% prevalence) may reduce the statistical power of our MR analyses. While we employed rigorous methods to mitigate this constraint, future replication in larger cohorts or meta-analyses is warranted to enhance the reliability of causal estimates. A further limitation of our study is the inability to perform colocalization analysis. This analysis is crucial for verifying that the genetic association between OSA and DN is driven by the same causal variant(s) rather than distinct variants in linkage disequilibrium, which would reduce the risk of confounding by genetic pleiotropy. The primary constraint preventing this analysis was the lack of sufficient sample overlap between the source GWAS for OSA and DN, which is a common issue when leveraging large, publicly available summary datasets from different consortia. Without access to individual-level data or matched summary statistics from the same cohort, reliable colocalization testing was not feasible. Therefore, while our MR analyses suggest a causal relationship, we cannot definitively rule out the possibility that distinct, correlated causal variants underlie the genetic associations for the exposure and outcome. Future studies with access to harmonized individual-level genetic data from cohorts with both phenotyped OSA and DN are warranted to perform colocalization and further solidify the causal inference. Furthermore, the short-term nature of typical clinical trials means that our study may not provide information on immediate intervention effects. This is crucial for questions that directly pertain to clinical interventions. While our research sheds light on the link between OSA and DN, further investigation is necessary to determine if treatment or interventions for OSA are necessary in real-world clinical settings, and how they should be implemented. Additional research may be needed to fully understand the implications of our findings in practical clinical scenarios.

Future research should validate these findings in prospective cohort studies, explore whether OSA treatment alters renal outcomes in interventional trials, and extend Mendelian randomization analyses to multi-ancestry populations to improve generalizability. Such work will be essential to determine whether OSA screening and management can be integrated into preventive strategies for DN.

## Supplementary Material

SNP information.docx

Revised Supplementary Tables.docx

02 multivariable mr analysis R.docx

03 power analysis R.docx

01 bidirectional mr analysis R.docx

## Data Availability

The raw data of this study were obtained from the GWAS public database https://gwas.mrcieu.ac.uk), and all data were freely downloaded and used. A variety of data analysis methods are freely available on the R platform.

## References

[1] KDOQI. KDOQI clinical practice guidelines and clinical practice recommendations for diabetes and chronic kidney disease. Am J Kidney Dis. 2007;49(2 Suppl 2):S12–S154. doi:10.1053/j.ajkd.2006.12.005. 17276798

[2] Afkarian M, Sachs MC, Kestenbaum B, et al. Kidney disease and increased mortality risk in type 2 diabetes. J Am Soc Nephrol. 2013;24(2):302–308. doi:10.1681/ASN.2012070718. 23362314 PMC3559486

[3] Fox CS, Matsushita K, Woodward M, et al. Associations of kidney disease measures with mortality and end-stage renal disease in individuals with and without diabetes: a meta-analysis. Lancet. 2012;380(9854):1662–1673. doi:10.1016/S0140-6736(12)61350-6. 23013602 PMC3771350

[4] Lu J, Zhao W, Chen T, et al. Influence of guideline adherence and parameter control on the clinical outcomes in patients with diabetic nephropathy. BMJ Open Diabetes Res Care. 2020;8(1):e001166. doi:10.1136/bmjdrc-2019-001166. PMC7368486 32675172

[5] Chan GC, Tang SC. Diabetic nephropathy: landmark clinical trials and tribulations. Nephrol Dial Transplant. 2016;31(3):359–368. doi:10.1093/ndt/gfu411. 25637638

[6] Yun KJ, Kim HJ, Kim MK, et al. Risk factors for the development and progression of diabetic kidney disease in patients with type 2 diabetes mellitus and advanced diabetic retinopathy. Diabetes Metab J. 2016;40(6):473–481. doi:10.4093/dmj.2016.40.6.473. 27766790 PMC5167712

[7] Neumiller JJ, St Peter WL, Shubrook JH. Type 2 diabetes and chronic kidney disease: an opportunity for pharmacists to improve outcomes. J Clin Med. 2024;13(5):1367. doi:10.3390/jcm13051367. 38592214 PMC10932148

[8] Foster GD, Sanders MH, Millman R, et al. Obstructive sleep apnea among obese patients with type 2 diabetes. Diabetes Care. 2009;32(6):1017–1019. doi:10.2337/dc08-1776. 19279303 PMC2681024

[9] Tahrani AA, Ali A, Raymond NT, et al. Obstructive sleep apnea and diabetic nephropathy: a cohort study. Diabetes Care. 2013;36(11):3718–3725. doi:10.2337/dc13-0450. 24062320 PMC3816897

[10] Liu Q, Chang X, Lian R, et al. Evaluation of bi-directional causal association between obstructive sleep apnoea syndrome and diabetic microangiopathy: a Mendelian randomization study. Front Cardiovasc Med. 2024;11:1340602. doi:10.3389/fcvm.2024.1340602. 38784169 PMC11112003

[11] Burgess S, Davey Smith G, Davies NM, et al. Guidelines for performing Mendelian randomization investigations: update for summer 2023. Wellcome Open Res. 2019;4:186. doi:10.12688/wellcomeopenres.15555.3. 32760811 PMC7384151

[12] Davies NM, Holmes MV, Davey Smith G. Reading Mendelian randomisation studies: a guide, glossary, and checklist for clinicians. BMJ. 2018;362:k601. doi:10.1136/bmj.k601. 30002074 PMC6041728

[13] Sekula P, Del Greco M F, Pattaro C, et al. Mendelian randomization as an approach to assess causality using observational data. J Am Soc Nephrol. 2016;27(11):3253–3265. doi:10.1681/ASN.2016010098. 27486138 PMC5084898

[14] Wang S, Jiang H, Qi H, et al. Association between periodontitis and temporomandibular joint disorders. Arthritis Res Ther. 2023;25(1):143. doi:10.1186/s13075-023-03129-0. 37550788 PMC10408055

[15] Skrivankova VW, Richmond RC, Woolf BAR, et al. Strengthening the reporting of observational studies in epidemiology using mendelian randomization: the STROBE-MR statement. JAMA. 2021;326(16):1614–1621. doi:10.1001/jama.2021.18236. 34698778

[16] Vujkovic M, Keaton JM, Lynch JA, et al. Discovery of 318 new risk loci for Type 2 diabetes and related vascular outcomes among 1.4 million participants in a multi-ancestry meta-analysis. Nat Genet. 2020;52(7):680–691. doi:10.1101/19012690. 32541925 PMC7343592

[17] Liao J, Zhang Y, Tang Z, et al. Causal relationships between peripheral immune cells and Alzheimer’s disease: a two-sample Mendelian randomization study. Neurol Sci. 2024;45(7):3117–3124. doi:10.1007/s10072-024-07324-y. 38267604

[18] Li P, Wang H, Guo L, et al. Association between gut microbiota and preeclampsia-eclampsia: a two-sample Mendelian randomization study. BMC Med. 2022;20(1):443. doi:10.1186/s12916-022-02657-x. 36380372 PMC9667679

[19] Sanderson E, Davey Smith G, Windmeijer F, et al. An examination of multivariable Mendelian randomization in the single-sample and two-sample summary data settings. Int J Epidemiol. 2019;48(3):713–727. doi:10.1093/ije/dyy262. 30535378 PMC6734942

[20] Burgess S, Davies NM, Thompson SG. Bias due to participant overlap in two-sample Mendelian randomization. Genet Epidemiol. 2016;40(7):597–608. doi:10.1002/gepi.21998. 27625185 PMC5082560

[21] Brion MJ, Shakhbazov K, Visscher PM. Calculating statistical power in Mendelian randomization studies. Int J Epidemiol. 2013;42(5):1497–1501. doi:10.1093/ije/dyt179. 24159078 PMC3807619

[22] Burgess S. Sample size and power calculations in Mendelian randomization with a single instrumental variable and a binary outcome. Int J Epidemiol. 2014;43(3):922–929. doi:10.1093/ije/dyu005. 24608958 PMC4052137

[23] Burgess S, Thompson SG. Interpreting findings from Mendelian randomization using the MR-Egger method. Eur J Epidemiol. 2017;32(5):377–389. doi:10.1007/s10654-017-0255-x. 28527048 PMC5506233

[24] Leong WB, Jadhakhan F, Taheri S, et al. The association between obstructive sleep apnea on diabetic kidney disease: A systematic review and meta-analysis. Sleep. 2016;39(2):301–308. doi:10.5665/sleep.5432. 26414891 PMC4712397

[25] Chiang J-F, Sun M-H, Chen K-J, et al. Association between obstructive sleep apnea and diabetic macular edema in patients with Type 2 diabetes. Am J Ophthalmol. 2021;226:217–225. doi:10.1016/j.ajo.2021.01.022. 33529585

[26] Wang G, Ouyang J, Li S, et al. The analysis of risk factors for diabetic nephropathy progression and the construction of a prognostic database for chronic kidney diseases. J Transl Med. 2019;17(1):264. doi:10.1186/s12967-019-2016-y. 31409386 PMC6693179

[27] Samsu N. Diabetic nephropathy: challenges in pathogenesis, diagnosis, and treatment. Biomed Res Int. 2021;2021(1):1497449. doi:10.1155/2021/1497449. 34307650 PMC8285185

[28] Carneiro-Barrera A, Amaro-Gahete FJ, Díaz-Román A, et al. Interdisciplinary weight loss and lifestyle intervention for obstructive sleep apnoea in adults: rationale, design and methodology of the INTERAPNEA study. Nutrients. 2019;11(9):2227. doi:10.3390/nu11092227. 31540168 PMC6770131

[29] Drel VR, Pacher P, Stevens MJ, et al. Aldose reductase inhibition counteracts nitrosative stress and poly (ADP-ribose) polymerase activation in diabetic rat kidney and high-glucose-exposed human mesangial cells. Free Radic Biol Med. 2006;40(8):1454–1465. doi:10.1016/j.freeradbiomed.2005.12.034. 16631535 PMC2225484

[30] McEvoy RD, Antic NA, Heeley E, et al. CPAP for prevention of cardiovascular events in obstructive sleep apnea. N Engl J Med. 2016;375(10):919–931. doi:10.1056/NEJMoa1606599. 27571048

[31] Qiu ZH, Luo YM, McEvoy RD. The Sleep Apnea Cardiovascular Endpoints (SAVE) study: implications for health services and sleep research in China and elsewhere. J Thorac Dis. 2017;9(8):2217–2220. doi:10.21037/jtd.2017.06.142. 28932508 PMC5594164

[32] Kohler M, Stradling JR. Mechanisms of vascular damage in obstructive sleep apnea. Nat Rev Cardiol. 2010;7(12):677–685. doi:10.1038/nrcardio.2010.145. 21079639

[33] Arnardottir ES, Mackiewicz M, Gislason T, et al. Molecular signatures of obstructive sleep apnea in adults: a review and perspective. Sleep. 2009;32(4):447–470. doi:10.1093/sleep/32.4.447. 19413140 PMC2663860

[34] Tahrani AA, Ali A, Raymond NT, et al. Obstructive sleep apnea and diabetic neuropathy: a novel association in patients with type 2 diabetes. Am J Respir Crit Care Med. 2012;186(5):434–441. doi:10.1164/rccm.201112-2135OC. 22723291 PMC3443800

[35] Fujii H, Kono K, Nakai K, et al. Oxidative and nitrosative stress and progression of diabetic nephropathy in type 2 diabetes. Am J Nephrol. 2010;31(4):342–352. doi:10.1159/000297290. 20224273

[36] Chen YJ, Quilley J. Fenofibrate treatment of diabetic rats reduces nitrosative stress, renal cyclooxygenase-2 expression, and enhanced renal prostaglandin release. J Pharmacol Exp Ther. 2008;324(2):658–663. doi:10.1124/jpet.107.129197. 17993607

[37] Rodrigues AM, Bergamaschi CT, Araújo RC, et al. Effects of training and nitric oxide on diabetic nephropathy progression in type I diabetic rats. Exp Biol Med (Maywood). 2011;236(10):1180–1187. doi:10.1258/ebm.2011.011005. 21930716

[38] Badran M, Abuyassin B, Golbidi S, et al. Uncoupling of vascular nitric oxide synthase caused by intermittent hypoxia. Oxid Med Cell Longev. 2016;2016(1):2354870. doi:10.1155/2016/2354870. 27840666 PMC5093285

[39] Luo B, Li Y, Zhu M, et al. Intermittent hypoxia and atherosclerosis: from molecular mechanisms to the therapeutic treatment (Retraction published 2024 Jan 9;2024:9763571. doi:10.1155/2024/9763571). Oxid Med Cell Longev. 2022;2022:1438470. doi:10.1155/2022/1438470. 35965683 PMC9365608

[40] Zhou L, Chen P, Peng Y, et al. Role of oxidative stress in the neurocognitive dysfunction of obstructive sleep apnea syndrome. Oxid Med Cell Longev. 2016;2016(1):9626831. doi:10.1155/2016/9626831. 27774119 PMC5059616

[41] Sifuentes-Franco S, Padilla-Tejeda DE, Carrillo-Ibarra S, et al. Oxidative stress, apoptosis, and mitochondrial function in diabetic nephropathy. Int J Endocrinol. 2018;2018:1875870–1875813. doi:10.1155/2018/1875870. 29808088 PMC5902001

[42] Liu T, Zhan Y, Wang Y, et al. Obstructive sleep apnea syndrome and risk of renal impairment: a systematic review and meta-analysis with trial sequential analysis. Sleep Breath. 2021;25(1):17–27. doi:10.1007/s11325-020-02090-5. 32440991 PMC7987709

[43] Altaf QA, Dodson P, Ali A, et al. Obstructive sleep apnea and retinopathy in patients with Type 2 diabetes. A longitudinal study. Am J Respir Crit Care Med. 2017;196(7):892–900. doi:10.1164/rccm.201701-0175OC. 28594570 PMC5649977

[44] Leong WB, Jadhakhan F, Taheri S, et al. Effect of obstructive sleep apnoea on diabetic retinopathy and maculopathy: a systematic review and meta-analysis. Diabet Med. 2016;33(2):158–168. doi:10.1111/dme.12817. 26031931

[45] Bottini P, Redolfi S, Dottorini ML, et al. Autonomic neuropathy increases the risk of obstructive sleep apnea in obese diabetics. Respiration. 2008;75(3):265–271. doi:10.1159/000100556. 17347559

[46] Muraki I, Wada H, Tanigawa T. Sleep apnea and type 2 diabetes. J Diabetes Investig. 2018;9(5):991–997. doi:10.1111/jdi.12823. PMC6123041 29453905

