# Supplementary material for: Causal relationship between obstructive sleep apnea and diabetic nephropathy: bidirectional and multivariable Mendelian randomization study
Source: Ren Fail. 2025 Oct 14;47(1):2569086. doi: 10.1080/0886022X.2025.2569086 (PMC12599006; doi:10.1080/0886022X.2025.2569086)
Supplement: Revised Supplementary Tables.docx [file IRNF_A_2569086_SM4649.docx]

**Supplementary Tables**

**Table S1.** Extracted SNPs for the exposure OSA based on a genome-wide significance threshold of 5E-06

|  | A1 | A2 | BETA | EAF | SE | P | R² | F statistic |
| --- | --- | --- | --- | --- | --- | --- | --- | --- |
| rs10507084 | T | C | 0.103 | 0.165 | 0.019 | 5.51E-08 | 0.00291 | 1391.7913 |
| rs10513713 | A | G | 0.075 | 0.274 | 0.016 | 1.52E-06 | 0.002227 | 1064.2529 |
| rs10906985 | A | G | 0.066 | 0.378 | 0.013 | 4.05E-07 | 0.002048 | 978.6739 |
| rs11075985 | A | C | 0.104 | 0.372 | 0.013 | 1.99E-15 | 0.005015 | 2403.4939 |
| rs114983649 | A | C | 0.154 | 0.042 | 0.03 | 3.66E-07 | 0.001932 | 922.9719 |
| rs115903639 | A | G | 0.193 | 0.014 | 0.042 | 4.87E-06 | 0.001005 | 479.6652 |
| rs116182122 | G | T | 0.309 | 0.013 | 0.06 | 2.27E-07 | 0.002391 | 1142.9967 |
| rs116420235 | T | C | -0.209 | 0.016 | 0.044 | 2.51E-06 | 0.00137 | 654.1163 |
| rs11704027 | G | A | 0.069 | 0.281 | 0.015 | 1.72E-06 | 0.001941 | 927.2435 |
| rs11774552 | T | C | -0.066 | 0.414 | 0.013 | 5.05E-07 | 0.002126 | 1016.0758 |
| rs12699649 | T | C | 0.063 | 0.366 | 0.013 | 2.90E-06 | 0.001835 | 876.7201 |
| rs142416325 | A | G | -0.347 | 0.012 | 0.067 | 2.09E-07 | 0.002902 | 1387.6475 |
| rs144160082 | G | A | 0.125 | 0.053 | 0.027 | 4.00E-06 | 0.001575 | 752.308 |
| rs145058358 | C | A | 0.158 | 0.056 | 0.032 | 5.58E-07 | 0.002616 | 1250.774 |
| rs146794654 | T | C | -0.202 | 0.019 | 0.043 | 1.90E-06 | 0.001532 | 731.893 |
| rs16861404 | A | G | 0.079 | 0.169 | 0.017 | 2.10E-06 | 0.001761 | 841.2919 |
| rs17212403 | A | G | 0.092 | 0.129 | 0.019 | 8.95E-07 | 0.001881 | 898.8482 |
| rs17513135 | T | C | 0.077 | 0.206 | 0.016 | 7.37E-07 | 0.001932 | 922.8717 |
| rs17547430 | G | A | 0.127 | 0.06 | 0.026 | 6.36E-07 | 0.001808 | 863.7793 |
| rs1885070 | G | A | 0.066 | 0.283 | 0.014 | 4.32E-06 | 0.001783 | 851.761 |
| rs1956376 | T | C | 0.069 | 0.435 | 0.013 | 1.59E-07 | 0.002341 | 1118.7593 |
| rs2277339 | G | T | -0.104 | 0.135 | 0.02 | 1.49E-07 | 0.002519 | 1204.1575 |
| rs2611742 | C | T | 0.063 | 0.422 | 0.013 | 1.30E-06 | 0.00193 | 922.2121 |
| rs4488813 | G | A | -0.106 | 0.132 | 0.023 | 4.50E-06 | 0.002577 | 1232.1102 |
| rs573593537 | C | T | 0.203 | 0.078 | 0.044 | 3.95E-06 | 0.005919 | 2839.3778 |
| rs58138946 | A | G | 0.17 | 0.044 | 0.037 | 4.05E-06 | 0.002411 | 1152.53 |
| rs6815442 | T | A | 0.069 | 0.315 | 0.015 | 2.53E-06 | 0.002036 | 972.7185 |
| rs7133573 | T | C | -0.061 | 0.568 | 0.013 | 2.44E-06 | 0.001844 | 880.8945 |
| rs72683533 | A | G | 0.166 | 0.045 | 0.036 | 4.75E-06 | 0.00236 | 1128.0639 |
| rs73156514 | G | C | 0.092 | 0.119 | 0.019 | 1.17E-06 | 0.001777 | 848.9069 |
| rs76054872 | T | G | 0.208 | 0.025 | 0.045 | 3.10E-06 | 0.002069 | 988.5804 |
| rs79672111 | A | G | 0.239 | 0.018 | 0.052 | 4.46E-06 | 0.002014 | 962.5003 |

OSA, obstructive sleep apnea; SNP, single-nucleotide polymorphism, A1, effect allele, A2, non-effect allele, EAF, Effect allele frequency, BETA, beta estimate for the association of SNP with exposure, SE, standard error of the Beta, P, two-sided P-value from the meta-analysis for exposure, R^2^ (%), explained variance given in percent.

**Table S2.** Extracted SNPs for the exposure DN based on a genome-wide significance threshold of 5E-6

| SNP | A1 | A2 | BETA | EAF | SE | P | R² | F statistic |
| --- | --- | --- | --- | --- | --- | --- | --- | --- |
| rs11629844 | T | A | 0.214 | 0.322 | 0.046 | 3.83E-06 | 0.019999 | 9229.6301 |
| rs116739291 | A | T | 1.222 | 0.047 | 0.261 | 2.88E-06 | 0.132986 | 69371.9472 |
| rs116884601 | C | A | 1.634 | 0.027 | 0.343 | 1.83E-06 | 0.138932 | 72974.4190 |
| rs12661002 | G | A | -0.190 | 0.454 | 0.041 | 3.97E-06 | 0.017934 | 8259.0482 |
| rs184896558 | T | C | 0.723 | 0.031 | 0.153 | 2.19E-06 | 0.030941 | 14440.7651 |
| rs2237897 | T | C | -0.324 | 0.249 | 0.068 | 2.20E-06 | 0.039120 | 18413.5283 |
| rs28564801 | C | T | 0.238 | 0.219 | 0.052 | 4.15E-06 | 0.019424 | 8958.9593 |
| rs35978445 | A | G | -0.211 | 0.473 | 0.042 | 5.80E-07 | 0.022193 | 10265.3007 |
| rs55853916 | C | G | 0.345 | 0.200 | 0.062 | 3.05E-08 | 0.038195 | 17960.9690 |
| rs58867595 | A | G | 0.405 | 0.141 | 0.082 | 7.29E-07 | 0.039580 | 18638.9479 |
| rs59156568 | T | A | 0.883 | 0.022 | 0.192 | 4.26E-06 | 0.032903 | 15387.5074 |
| rs73776960 | T | C | 0.749 | 0.026 | 0.158 | 2.24E-06 | 0.028949 | 13483.5431 |
| rs76441909 | T | C | 0.724 | 0.024 | 0.157 | 3.82E-06 | 0.024253 | 11241.7494 |
| rs76520075 | G | A | 0.489 | 0.057 | 0.106 | 4.07E-06 | 0.025929 | 12039.0703 |
| rs79535930 | A | G | 0.723 | 0.015 | 0.155 | 2.84E-06 | 0.015216 | 6988.2045 |

DN, Diabetic Nephropathy; SNP, single-nucleotide polymorphism; A1, effect allele; A2, non-effect allele; EAF, effect allele frequency; BETA, beta estimate for the association of SNP with exposure; SE, standard error of the Beta; P, two-sided P-value from the meta-analysis for exposure, R^2^ (%), explained variance (%).

**Table S3.** Results of the Wald ratio analysis using the SNP meeting the genome-wide significance threshold (P < 5×10⁻⁸)

| **Variable** | **Value** |
| --- | --- |
| Outcome | Diabetic nephropathy |
| Exposure | Sleep apnea syndrome |
| Method | Wald ratio |
| No. of SNPs | 1 |
| Beta | 0.89 |
| SE | 0.41 |
| P-value | 0.029 |
| 95% CI | 0.09 to 1.70 |
| Odds Ratio (OR) | 2.45 |
| 95% CI (OR) | 1.09 to 5.48 |

|  |
| --- |

SNP, single nucleotide polymorphism; SE, standard error; CI, confidence interval; OR, odds ratio;Beta, beta coefficient.

**FigureS1**: The scatter plot for MR analyses of causal associations between each DN related SNP and OSA.


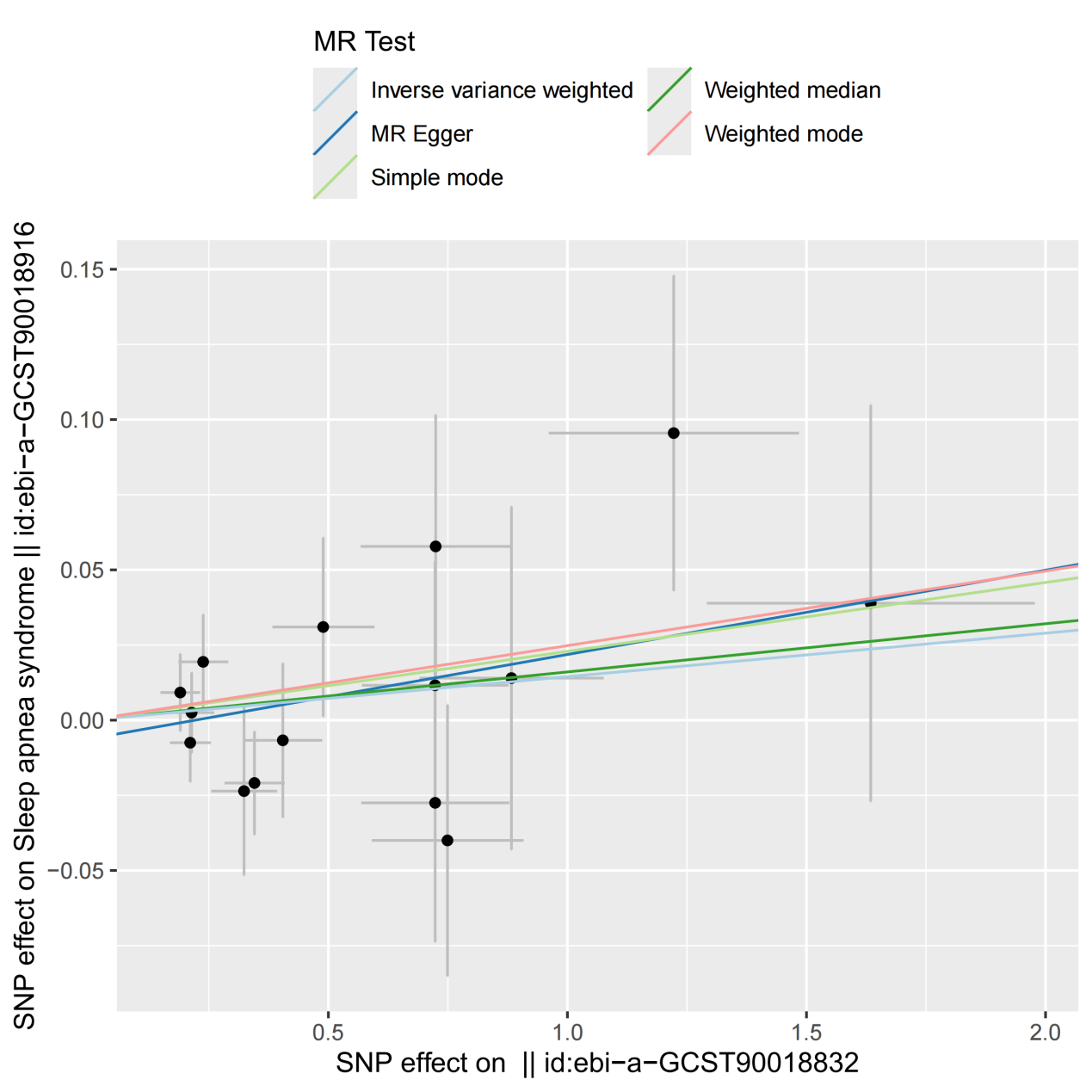


OSA, Obstructive sleep apnea; DN, diabetic nephropathy; SNP, single-nucleotide polymorphism.

**Figure S2.** Leave-one-out sensitivity analysis for DN on OSA.


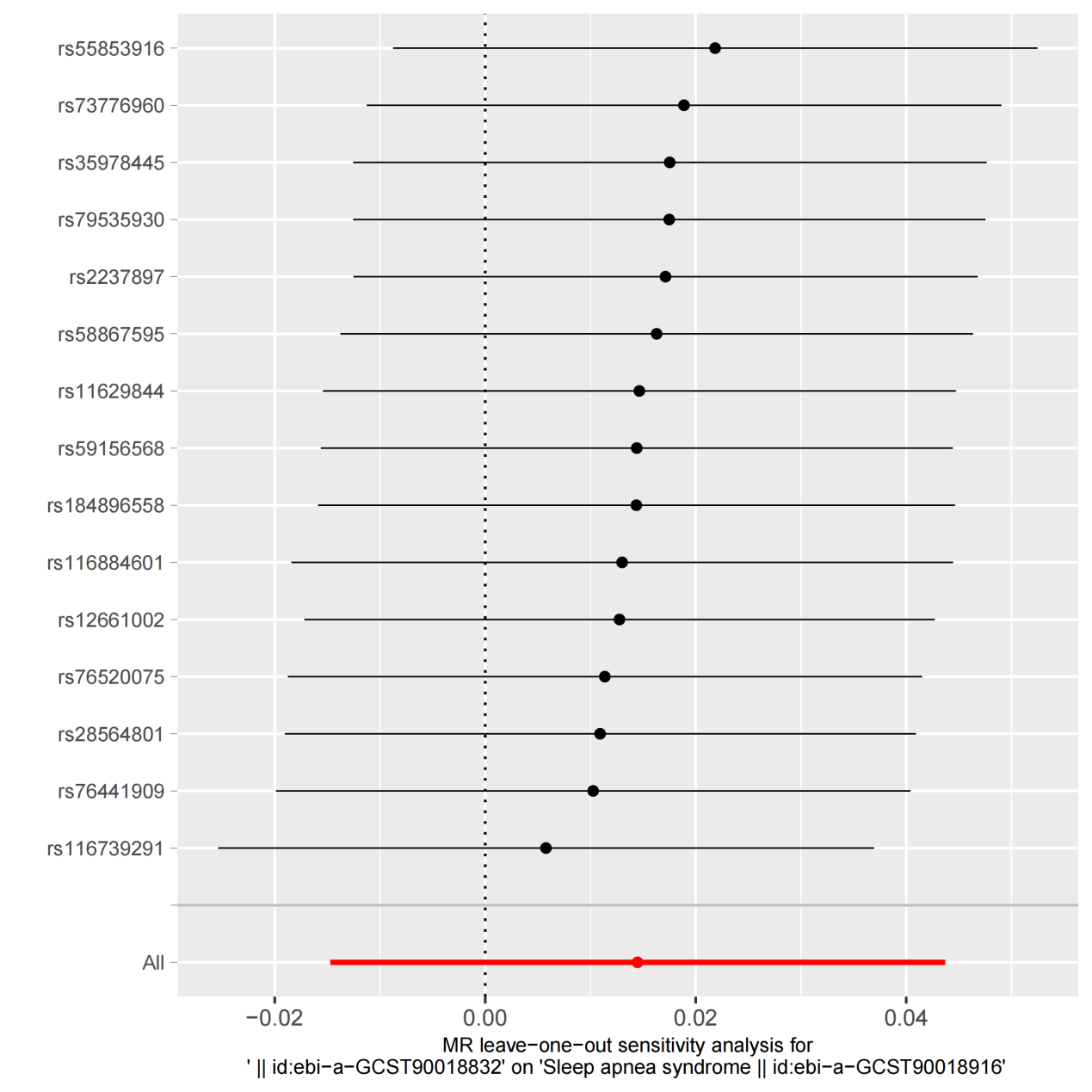


OSA, Obstructive sleep apnea; DN, diabetic nephropathy; SNP, single-nucleotide polymorphism.

**Figure S3.** Funnel plots of DN against the genetic associations with OSA


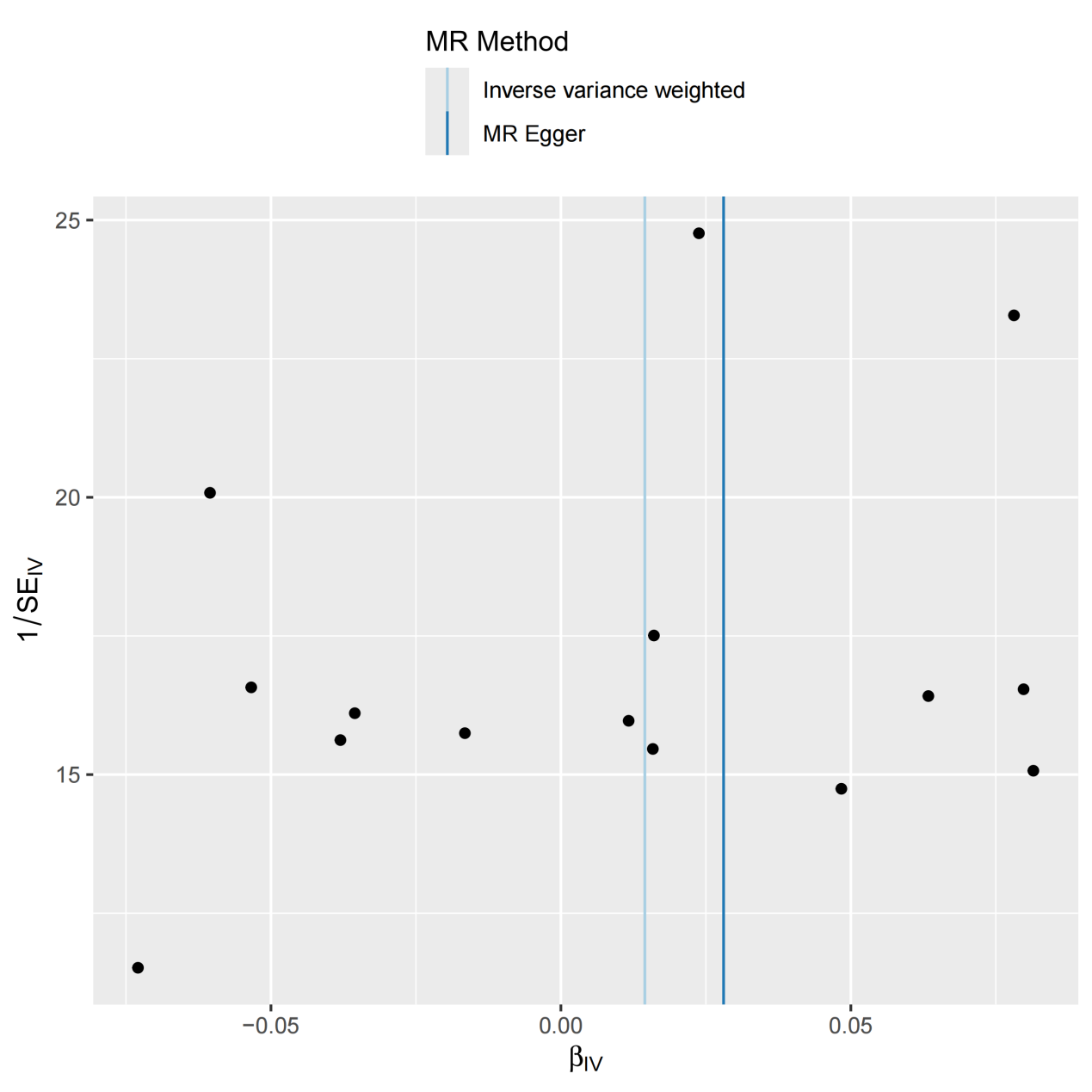


OSA, Obstructive sleep apnea; DN, diabetic nephropathy; SNP, single-nucleotide polymorphism.

**Figure S4.** Funnel plots of OSA against the genetic associations with DN.


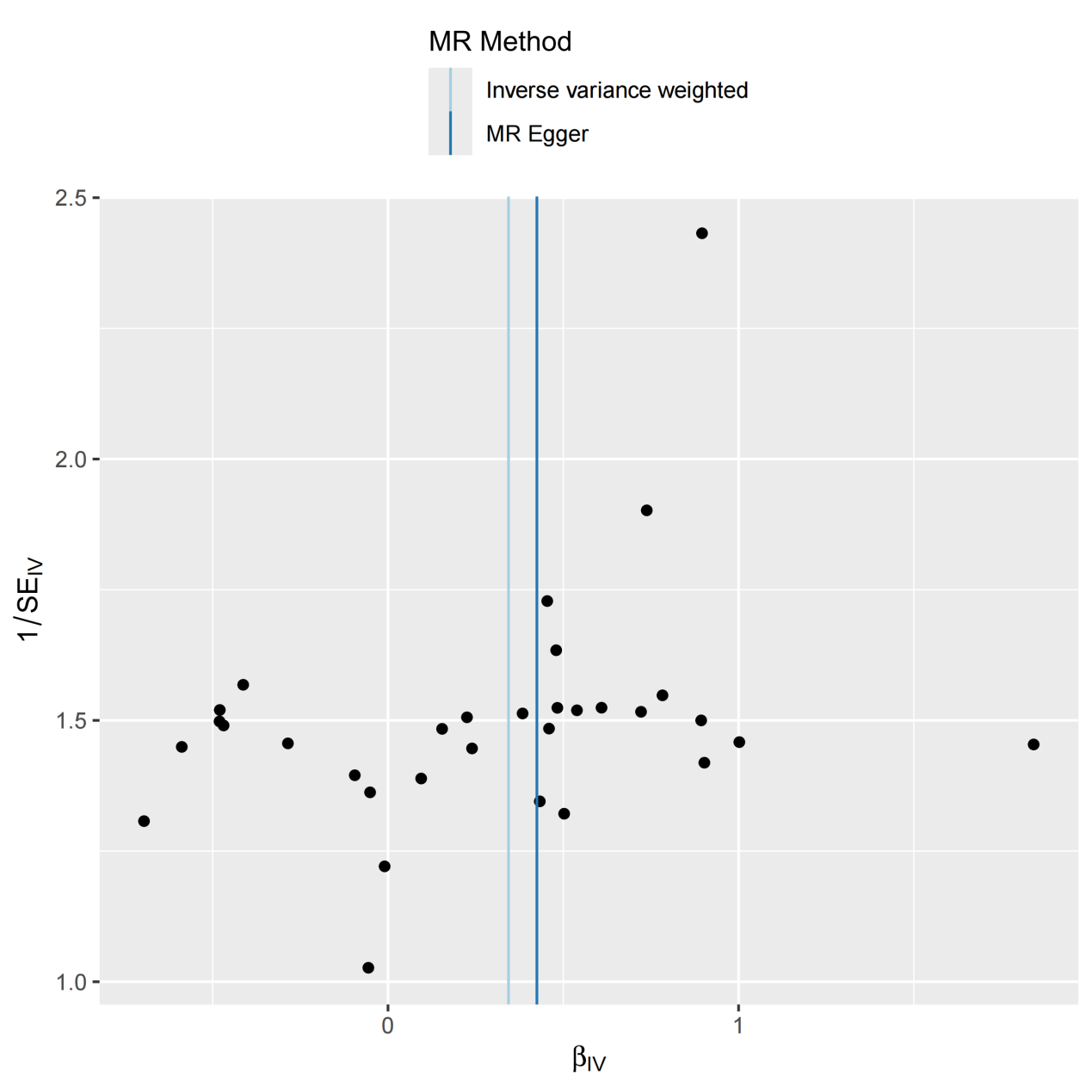


OSA, Obstructive sleep apnea; DN, diabetic nephropathy; SNP, single-nucleotide polymorphism.

**Figure S5.** Scatterplot of the genetic associations for the SNP instrument with the exposure (OSA) and the outcome (DN).


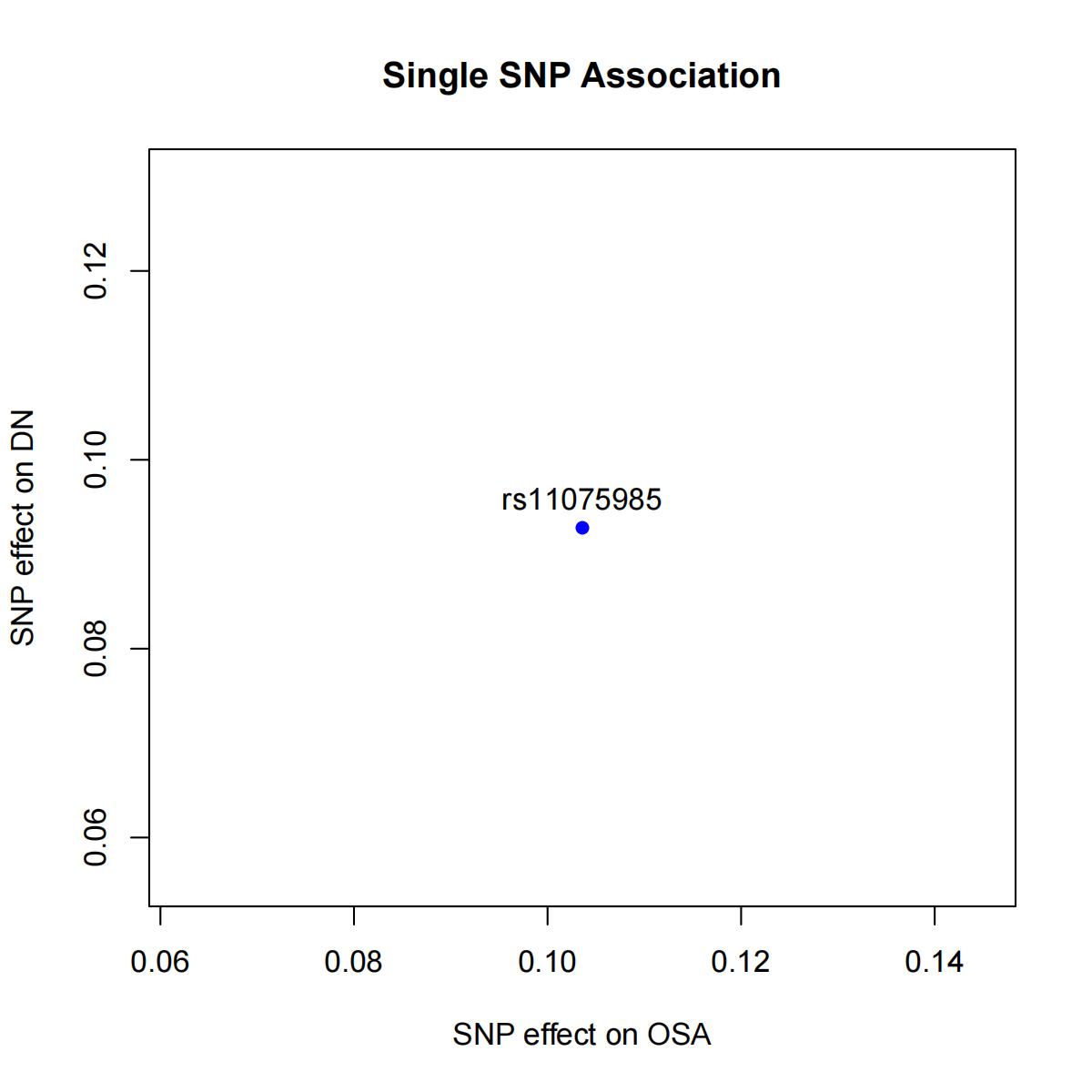


OSA, Obstructive sleep apnea; DN, diabetic nephropathy; SNP, single-nucleotide polymorphism.
