# Supplementary material for: Causal relationship between obstructive sleep apnea and diabetic nephropathy: bidirectional and multivariable Mendelian randomization study
Source: Ren Fail. 2025 Oct 14;47(1):2569086. doi: 10.1080/0886022X.2025.2569086 (PMC12599006; doi:10.1080/0886022X.2025.2569086)
Supplement: 02 multivariable mr analysis R.docx [file IRNF_A_2569086_SM4648.docx]

####多变量孟德尔随机化，线上提取数据操作方法####

#多变量MR

#remotes::install_github("WSpiller/RMVMR", build_opts = c("--no-resave-data","--no-manual"), build_vignettes = TRUE)

#安装MendelianRandomization包，最简单

#install.packages("MendelianRandomization")

#读取本地数据的vCfR包

library(RMVMR)

library(MendelianRandomization)

#读取本地数据的包

#install.packages("data.table")

library(data.table)

#加载包

library(TwoSampleMR)

library(ieugwasr)

Sys.setenv(OPENGWAS_JWT="eyJhbGciOiJSUzI1NiIsImtpZCI6ImFwaS1qd3QiLCJ0eXAiOiJKV1QifQ.eyJpc3MiOiJhcGkub3Blbmd3YXMuaW8iLCJhdWQiOiJhcGkub3Blbmd3YXMuaW8iLCJzdWIiOiJsenIxNzgzNTcwMTU5MUBvdXRsb29rLmNvbSIsImlhdCI6MTc1NzY5MTcwOSwiZXhwIjoxNzU4OTAxMzA5fQ.o_SXxw-UzE0i6lU1JkFmMaI-soQbrvYYjIEmG69LjQiqpAwDIIFKSUawQcQiJ-cU7gLcQbK7yy8-w736KKv-v6E69HpP_daj_FYKAzaWxCNCPkbk55v2xnZ2kBQABxQCmL7unYfJGayTOZgH6Yus2nF6wUeeLQ0FUWVtl8vs1XZTt_UhPJFAFTVQx9K1cSgvb5avUMAACBkelfikO1Ni8rQlQrfcCDPfm1C-I7sot0ZjNwp0u9PNMtvs2JueWrFgl7eXm6ru95k2phpmkBKjiqNqUfAjQ3809Qc4Pcxi4T-CSW6WN2MQVLc-PK8VYSzE7tJgDfsveIiAkQx2z2074g")

####IEU在线提取数据MVMR####

#以下四个暴露分别是高血脂 高血压 OSA

id_exp <- c("ebi-a-GCST90104003","ebi-a-GCST90038608","ebi-a-GCST90018916")

#三个暴露因素SNP提取，只要有一个强相关的SNP都提取

exp_dat <- mv_extract_exposures(id_exp)

#保存数据

write.csv(exp_dat,file="exp_dat_onlin.csv")

#线上读取不了，备用方法

# exp_dat<-read.csv("exp_dat_onlin.csv")

#IEU中结局ID

id_out <- "ebi-a-GCST90018832"

#根据暴露的SNP，提取结局数据

out_dat <- extract_outcome_data(exp_dat$SNP, id_out)

#保存数据

write.csv(out_dat,file="out_dat_onlin.csv")

# #线上读取不了，备用方法

# out_dat<-read.csv("out_dat_onlin.csv")

#协调暴露与结局的数据,线上数据一次性协调

mvdat <- mv_harmonise_data(exp_dat, out_dat)

#使用IVW进行合并分析，TwoSampleMR包只能进行IVW

res <- mv_multiple(mvdat)

res

#计算OR值及可信区间

res_OR<-generate_odds_ratios(res$result)

res_OR

####传统的MR，与多变量的比较####

# #提取单个暴露因素的snp

# exp_HDL <- extract_instruments(

# outcomes = 'ieu-a-299'

# )

#

# #根据暴露的SNP，提取结局数据

# out_CVD <- extract_outcome_data(

# snps = exp_HDL$SNP,

# outcomes = 'ieu-a-7')

#

# #协调效应，合并数据

# dat_HDL <- harmonise_data(

# exposure_dat = exp_HDL,

# outcome_dat = out_CVD

# )

#

# #用IVW方法,进行mr分析

# res_HDL <- mr(dat_HDL,method_list = c("mr_ivw"))

# res_HDL

#

#

# #呈现OR值及95%可信区间，如果只呈现beta值和95%可信区间也可以用这个代码

# generate_odds_ratios(res_HDL)

####更多的多变量MR方法####

#利用MendelianRandomization进行MR-Egger，Median分析

library(MendelianRandomization)

#MendelianRandomization #需要对数据进行重新构建

mendedata <- mr_mvinput(bx = mvdat$exposure_beta, bxse = mvdat$exposure_se,

by = mvdat$outcome_beta, byse = mvdat$outcome_se,

correlation =matrix(),

exposure = c(" HDL","LDL","TG"),outcome = "CVD")

#IVW

res_ivw<-mr_mvivw(mendedata)

res_ivw

#MR-Egger

res_ergger<-mr_mvegger(mendedata)

res_ergger

#Median

res_median<-mr_mvmedian(mendedata)

res_median

# 如何把这个MendelianRandomization结果改成TwoSampleMR的格式：

# 首先运行您的 MendelianRandomization 分析

library(MendelianRandomization)

# 假设 mvdat 是您的数据

mendedata <- mr_mvinput(bx = mvdat$exposure_beta, bxse = mvdat$exposure_se,

by = mvdat$outcome_beta, byse = mvdat$outcome_se,

exposure = c("HDL", "LDL", "TG"), outcome = "CVD")

# MR-Egger

res_egger <- mr_mvegger(mendedata)

# 将结果转换为 TwoSampleMR 格式的函数

convert_to_twosamplemr_format <- function(mr_result, method_name, exposure_names) {

result <- data.frame(

id.exposure = exposure_names,

exposure = paste0(exposure_names, " || method:", method_name),

id.outcome = "Outcome",

outcome = "CVD",

nsnp = mr_result@SNPs,

b = mr_result@Estimate,

se = mr_result@StdError,

pval = mr_result@Pvalue,

stringsAsFactors = FALSE

)

# 计算置信区间

result$lo_ci <- result$b - 1.96 * result$se

result$up_ci <- result$b + 1.96 * result$se

return(result)

}

# 转换 MR-Egger 结果

egger_formatted <- convert_to_twosamplemr_format(res_egger, "MR-Egger", c("HDL", "LDL", "TG"))

# 计算 OR 值

egger_formatted$or <- exp(egger_formatted$b)

egger_formatted$or_lci95 <- exp(egger_formatted$lo_ci)

egger_formatted$or_uci95 <- exp(egger_formatted$up_ci)

# 查看格式化后的结果

print(egger_formatted)

# 保存结果到CSV文件

write.csv(egger_formatted, "mr_egger_results_formatted.csv", row.names = FALSE)

cat("MR-Egger 结果已保存至: mr_egger_results_formatted.csv\n")

#敏感性分析

#Obtain data

#Format data

#Assess instrument strength

#Assess horizontal pleiotropy

#Estimate causal effects

#需要用到MVMR包，加载MVMR包

library(MVMR)

#数据的重新构建

MVMR_dat <- format_mvmr(BXGs = mvdat$exposure_beta,

BYG = mvdat$outcome_beta,

seBXGs = mvdat$exposure_se,

seBYG = mvdat$outcome_se,

RSID = rownames(mvdat$exposure_beta))

head(MVMR_dat)

#工具变量的强弱，计算snp的强度

F1 <- strength_mvmr(r_input = MVMR_dat, gencov = 0)

F2 <- strength_mvmr(r_input = mendedata, gencov = 0)

#异质性检验

mv_hete1 <- pleiotropy_mvmr(r_input = MVMR_dat, gencov = 0)

mv_hete2 <- pleiotropy_mvmr(r_input = mendedata, gencov = 0)

#水平多效性

#加载水平多效性包

library(MRPRESSO)

mr_presso(BetaOutcome = "betaYG",

BetaExposure = c("betaX1", "betaX2","betaX3"),

SdOutcome = "sebetaYG",

SdExposure = c("sebetaX1", "sebetaX2","sebetaX3"),

OUTLIERtest = TRUE,

DISTORTIONtest = TRUE,

data = MVMR_dat,

NbDistribution = 1000,

SignifThreshold = 0.05)

#暴露的筛选，TwoSampleMR包

mv_lasso_feature_selection(mvdat) #LASSO

mv_lasso<-mv_subset(

mvdat,

features = mv_lasso_feature_selection(mvdat), #LASSO后进行MR

intercept = FALSE,

instrument_specific = F,

pval_threshold = 5e-08,

plots = T

)

mv_lasso

mv_lasso$plots[[1]]

mv_lasso$plots[[2]]

mv_lasso$plots[[3]]

mv_lasso_OR<-generate_odds_ratios(mv_lasso$result)

mv_lasso_OR
