# Supplementary material for: Causal relationship between obstructive sleep apnea and diabetic nephropathy: bidirectional and multivariable Mendelian randomization study
Source: Ren Fail. 2025 Oct 14;47(1):2569086. doi: 10.1080/0886022X.2025.2569086 (PMC12599006; doi:10.1080/0886022X.2025.2569086)
Supplement: 03 power analysis R.docx [file IRNF_A_2569086_SM4647.docx]

############################################

## Mendelian Randomization Power Functions ##

############################################

# ---------- 辅助量 ----------

.f_stat <- function(N, R2) N * R2 / (1 - R2) # F-statistic

.chi_thr <- function(alpha) qchisq(1 - alpha, df = 1) # 卡方阈值

# ---------- 1) 结局为二分类 ----------

# N : 样本量（给定功效时设 NA）

# power: 期望功效（给定样本量时设 NA）

# alpha: I 型错误率

# R2 : IV 对暴露解释度

# K : 结局流行率 (Pr[Y = 1])

# OR : MR 效应 (单位暴露对结局的 OR)

mr_power_binary <- function(N = NA, power = NA,

alpha = 0.05,

R2, K, OR) {

stopifnot(xor(is.na(N), is.na(power))) # 二选一

thr <- .chi_thr(alpha)

# 真实风险差 (risk difference) 的近似换算

b_MR <- K * (OR / (1 + K * (OR - 1)) - 1)

if (is.na(power)) { # --- 给定 N 求功效 ---

v_MR <- (K * (1 - K) - b_MR^2) / (N * R2)

ncp <- b_MR^2 / v_MR # 非中心参数

pow <- 1 - pchisq(thr, df = 1, ncp = ncp)

data.frame(Parameter = c("Power", "NCP", "F-statistic"),

Value = c(pow, ncp, .f_stat(N, R2)),

Description= c("", "Non-Centrality Parameter",

"Instrument strength (F)"))

} else { # --- 给定功效求 N ---

z1 <- qnorm(1 - alpha / 2)

z2 <- qnorm(power)

Z <- (z1 + z2)^2

f <- K * (1 - K) - b_MR^2

N <- ceiling(Z * f / (b_MR^2 * R2))

data.frame(Parameter = "Sample Size", Value = N)

}

}

# ---------- 2) 结局为连续型 ----------

# byx : MR 效应估计 (β_YX)

# bOLS: 观察性 β̂ (若无可设 0)

# varx: 暴露方差

# vary: 结局方差

mr_power_continuous <- function(N = NA, power = NA,

alpha = 0.05,

R2, byx, bOLS,

varx, vary) {

stopifnot(xor(is.na(N), is.na(power))) # 二选一

thr <- .chi_thr(alpha)

# 观测暴露-结局混杂贡献

con <- (bOLS - byx) * varx

vey <- vary - byx * varx * (2 * bOLS - byx)

if (vey < 0) stop("参数组合导致残差方差 < 0，请检查输入。")

if (is.na(power)) { # --- 给定 N 求功效 ---

b2sls <- byx + con / (N * R2)

v2sls <- vey / (N * R2 * varx)

ncp <- b2sls^2 / v2sls

pow <- 1 - pchisq(thr, df = 1, ncp = ncp)

data.frame(Parameter = c("Power", "NCP", "F-statistic"),

Value = c(pow, ncp, .f_stat(N, R2)),

Description= c("", "Non-Centrality Parameter",

"Instrument strength (F)"))

} else { # --- 给定功效求 N ---

z1 <- qnorm(1 - alpha / 2)

z2 <- qnorm(power)

Z <- (z1 + z2)^2

# 解一元二次方程 aN² + bN + c = 0

a <- (byx * R2)^2

b <- R2 * (2 * byx * con - Z * vey / varx)

c <- con^2

disc <- b^2 - 4 * a * c

if (disc < 0) stop("无实数解，请检查输入或降低目标功效。")

Nsol <- ceiling((-b + sqrt(disc)) / (2 * a))

data.frame(Parameter = "Sample Size", Value = Nsol)

}

}

############################################

## —— End of file —— ##

############################################

# 结局为二分类：已知样本量想算功效

mr_power_binary(N = 150000,

R2 = 0.02,

K = 0.10,

OR = 1.15)

# 结局为连续型：需要多少样本才能达 80% 功效

mr_power_continuous(power = 0.80,

R2 = 0.03,

byx = 0.05,

bOLS = 0.07,

varx = 1,

vary = 1.2)
