# Supplementary material for: Causal relationship between obstructive sleep apnea and diabetic nephropathy: bidirectional and multivariable Mendelian randomization study
Source: Ren Fail. 2025 Oct 14;47(1):2569086. doi: 10.1080/0886022X.2025.2569086 (PMC12599006; doi:10.1080/0886022X.2025.2569086)
Supplement: 01 bidirectional mr analysis R.docx [file IRNF_A_2569086_SM4646.docx]

#设置工作环境

setwd("F:\\科研\\2020EULAR\\结果\\多发性肌炎与皮肌炎") #快捷键：shift + ctrl + H

#打开R包

rm(list = ls()) #清空环境，数据多了容易混淆

Sys.setenv(LANGUAGE = "en")

options(stringsAsFactors = FALSE)

#install.packages("remotes") #安装包

#remotes::install_github("MRCIEU/TwoSampleMR")

library(TwoSampleMR)

#读取暴露数据-----------------------------

a <- seq(1:731) #根据需要改a和b的信息

b <- paste("ebi-a-GCST",90001390 + a,sep = "")

exp_dat_clumped = extract_instruments(outcomes = "ebi-a-GCST90018916", p1 = 5e-6, clump=TRUE, r2=0.001, kb=10000)

#exp_dat_clumped6$samplesize.exposure <- 171748 #看情况选择输入样本量

#计算R2和F值--------------

exp_dat_clumped$r2 = 2*(1-exp_dat_clumped$eaf.exposure)*(exp_dat_clumped$eaf.exposure)*(exp_dat_clumped$beta.exposure)^2

exp_dat_clumped$F = (exp_dat_clumped$r2/(1-exp_dat_clumped$r2))*(exp_dat_clumped$samplesize.exposure-2)

exp_dat_clumped = exp_dat_clumped[exp_dat_clumped$F>=10, ]

exp_dat_clumped = exp_dat_clumped[exp_dat_clumped$eaf.exposure>=0.01, ]

View(exp_dat_clumped)

#总F

F_all = (sum(exp_dat_clumped$r2)/(1-sum(exp_dat_clumped$r2)))*(unique(exp_dat_clumped$samplesize.exposure)-length(exp_dat_clumped$samplesize.exposur)-1)/length(exp_dat_clumped$samplesize.exposur)

#如果上面内容跑完数据掉了很多，就先不计算R2和F值了，这段不跑

#读取outcome数据（基于去掉混淆因素后的结果）-------------------

outcome_dat <- extract_outcome_data(snps = exp_dat_clumped$SNP,outcomes = "ebi-a-GCST90018832")

#Harmonize data

dat <- harmonise_data(exposure_dat = exp_dat_clumped,outcome_dat = outcome_dat, action = 2)

#phenoscanner找混杂SNP(先不跑)

#dat <- dat[-c(1,2,3),]#根据实际情况

table(dat$pval.outcome>1e-5) #如果有false的结果，要去掉

#第三个地方：去掉在结局比暴露中显著的snp位点--------------

View(dat[, c("pval.exposure", "pval.outcome")])

#dat=dat[-200,]#根据实际情况

write.csv(dat, file="SNP_information.csv")

#dat <- fread("dat_p_5_-8_ALL.csv")

#write.csv("mr_base_harmonised_data (1).csv", header = T, row.names = 1) 读取网站数据

#Perform MR--------------------------------------

#mr_report(dat) #自动生成报告

mr(dat) #MR默认五种算法

#mr(dat,method_list = c("mr_ivw","mr_egger_regression","mr_weighted_median")) #自行选择方法

pdf(file = "mr_scatter_plot_ALL.pdf",width = 6,height = 6)

mr_scatter_plot(mr_results = mr(dat,method_list = c("mr_egger_regression","mr_weighted_median","mr_ivw","mr_simple_mode","mr_weighted_mode")),dat)

dev.off()

generate_odds_ratios(mr_res = mr(dat, method_list = c("mr_egger_regression","mr_weighted_median","mr_ivw","mr_simple_mode","mr_weighted_mode"))) #转化成OR二分类

result <- generate_odds_ratios(mr_res = mr(dat, method_list = c("mr_egger_regression","mr_weighted_median","mr_ivw","mr_simple_mode","mr_weighted_mode"))) #转化成OR二分类

write.csv(mr_result,"mr_result.csv") #保存数据

# 使用Wald比率法计算OR值（适用于单SNP分析）

mr_result <- mr(dat, method_list = "mr_wald_ratio")

mr_result <- generate_odds_ratios(mr_result)

print(mr_result)

# 或者简单绘制效应大小图（非标准散点图）

pdf(file = "single_snp_effect.pdf", width = 6, height = 6)

plot(dat$beta.exposure, dat$beta.outcome,

xlab = "SNP effect on OSA",

ylab = "SNP effect on DN",

main = "Single SNP Association",

pch = 16, col = "blue")

abline(h = 0, v = 0, lty = 2)

text(dat$beta.exposure, dat$beta.outcome,

labels = dat$SNP, pos = 3)

dev.off()

#单个SNP森林图

pdf(file = "mr_singlesnp_plot_ALL.pdf",width = 6,height = 6)

res_single <- mr_singlesnp(dat)

mr_forest_plot(res_single)

dev.off()

#异质性检测(注Q值的大小，如果Q>0.1表明没有异质性)

mr_heterogeneity(dat)

#异质性可视化

pdf(file = "mr_funnel_plot_1e-5_ALL.pdf",width = 6,height = 6)

mr_funnel_plot(singlesnp_results = mr_singlesnp(dat))

dev.off()

#多效性检测

mr_pleiotropy_test(dat)

#Leave-one-out analysis

pdf(file = "mr_leaveoneout_plot_1e-5_ALL.pdf",width = 6,height = 6)

mr_leaveoneout_plot(leaveoneout_results = mr_leaveoneout(dat))

dev.off()
